# Induced CD8**α** identifies human NK cells with enhanced proliferative fitness and modulates NK cell activation

**DOI:** 10.1172/JCI173602

**Published:** 2024-05-28

**Authors:** Celia C. Cubitt, Pamela Wong, Hannah K. Dorando, Jennifer A. Foltz, Jennifer Tran, Lynne Marsala, Nancy D. Marin, Mark Foster, Timothy Schappe, Hijab Fatima, Michelle Becker-Hapak, Alice Y. Zhou, Kimberly Hwang, Miriam T. Jacobs, David A. Russler-Germain, Emily M. Mace, Melissa M. Berrien-Elliott, Jacqueline E. Payton, Todd A. Fehniger

**Affiliations:** 1 Division of Oncology, Siteman Cancer Center, and; 2 Department of Pathology and Immunology, Washington University School of Medicine, St. Louis, Missouri, USA.; 3 Division of Allergy, Immunology and Rheumatology, Department of Pediatrics, Columbia University Vagelos College of Physicians and Surgeons, New York, New York, USA.

**Keywords:** Immunology, Cancer, Innate immunity, NK cells

## Abstract

The surface receptor CD8α is present on 20%–80% of human (but not mouse) NK cells, yet its function on NK cells remains poorly understood. CD8α expression on donor NK cells was associated with a lack of therapeutic responses in patients with leukemia in prior studies, thus, we hypothesized that CD8α may affect critical NK cell functions. Here, we discovered that CD8α^–^ NK cells had improved control of leukemia in xenograft models compared with CD8α^+^ NK cells, likely due to an enhanced capacity for proliferation. Unexpectedly, we found that CD8α expression was induced on approximately 30% of previously CD8α^–^ NK cells following IL-15 stimulation. These induced CD8α^+^ (iCD8α^+^) NK cells had the greatest proliferation, responses to IL-15 signaling, and metabolic activity compared with those that sustained existing CD8α expression (sustained CD8α^+^) or those that remained CD8α^–^ (persistent CD8α^–^). These iCD8α^+^ cells originated from an IL-15Rβ^hi^ NK cell population, with CD8α expression dependent on the transcription factor RUNX3. Moreover, *CD8A* CRISPR/Cas9 deletion resulted in enhanced responses through the activating receptor NKp30, possibly by modulating KIR inhibitory function. Thus, CD8α status identified human NK cell capacity for IL-15–induced proliferation and metabolism in a time-dependent fashion, and its presence had a suppressive effect on NK cell–activating receptors.

## Abstract 2

CD8α impairs activation through NK receptors, and its expression on human NK cells marks capacity for IL-15–induced proliferation, metabolism, and functionality.

## Introduction

NK cells are innate lymphoid cells that protect the host from infection and malignant transformation through direct cytotoxicity and communication via cytokine and chemokine production (1–3). Human NK cells are categorized according to 2 distinct subsets in human peripheral blood: the immature and highly proliferative CD56^bright^ subset and the mature, less proliferative and more cytotoxic CD56^dim^ subset (1, 4). NK cell function is tightly regulated by a balance of germ-line DNA-encoded activating, inhibitory, and cytokine receptors (5, 6). The primary inhibitory receptors in human NK cells that promote self-tolerance include the killer cell Ig-like receptors (KIRs) that bind to MHC class I and the CD94-NKG2A receptor that recognizes the nonpolymorphic human leukocyte antigen-E (HLA-E) (7–9). NK cells also express multiple activating receptors that trigger effector functions when engaged, including the natural cytotoxicity receptors (NCRs) NKp30 and NKp46, the C-type lectin NKG2D, and other coreceptors such as CD16, CD2, and 2B4 (4, 5, 10). Although signaling through these receptors has been described to occur by associating with a variety of shared (CD16 and NKp30: CD3z/FcRγ) and distinct (NKG2D: DAP10; 2B4: SAP and Fyn; CD2: p56lck) signaling adaptors, additional mechanisms that modulate these signals are not clearly understood (10–13).

NK cells are particularly dependent on IL-15 signaling for their proliferation and survival (14–16). The IL-15 receptor is composed of 3 subunits: IL-2/15Rα (CD25), IL-15Rβ (CD122), and the shared common γ (CD132) chain. The IL-15 receptor signals via 3 distinct pathways: JAK1,-3/STAT5 (STAT5), Ras/Raf/Mek/Erk (MAPK), and PI3K/Akt/mTOR (AKT/mTOR), which drive transcriptional and metabolic programs that control NK homeostasis and proliferation (14, 17, 18). In particular, mTOR activation leads to translation initiation (by phosphorylating ribosomal protein 6 [S6]) and modulates metabolism via the upregulation of nutrient receptors and proteins involved in glycolysis and lipid synthesis (19, 20). Supporting this, studies in murine NK cells have established that glucose metabolism is essential for IL-15–driven proliferation (21, 22).

The biology of the coreceptor CD8α on human NK cells is not well understood, and mouse NK cells do not express CD8α (23). On average, 40% (range, 15%–88%) of human NK cells express the homodimeric CD8α receptor, and a small proportion (1%–2%) express the CD8αβ heterodimer (24, 25). While CD8αβ has been extensively characterized on T cells as a coreceptor for the T cell receptor (TCR), CD8αα is expressed on other immune cells, including intraepithelial lymphocytes, human monocytes, and murine DCs (26–28). CD8αα contains an extracellular region that can bind to the conserved a3 region of HLA class I and most nonclassical HLAs (except human HLA-E, due to a3 domain polymorphisms; refs. 29, 30), a transmembrane domain, and a cytoplasmic tail that associates with the Src tyrosine kinase Lck (26). There are limited and conflicting data on the function of CD8α in the biology of human NK cells. Previous studies have described that CD8α^+^ NK cells are more cytotoxic and mediate leukemia cell killing in patients who received autologous hematopoietic cell transplants (HCTs), although this study compared CD8α^+/–^ NK cells without accounting for the higher expression of CD8α on the more mature and cytotoxic CD56^dim^ subset (31). In patients with untreated chronic HIV infections, higher frequencies of CD8α^+^ NK cells were correlated with slower disease progression, while a CD8^+^ NK transcriptomic signature was associated with reduced relapse risk in patients with relapsing-remitting multiple sclerosis (32, 33). Additional conflicting effects have also been reported, with CD8α protecting NK cells from activation-induced apoptosis in one study, and CD8α engagement with soluble HLA class I triggering apoptosis in another (34, 35). A more recent study proposed that CD8α could facilitate NK cell licensing by binding to HLA class I and enhancing KIR binding (36).

Allogenic NK cellular immunotherapies have been investigated for treating cancer in multiple clinical trials (37, 38). Our previous work showed that NK cells briefly stimulated with IL-12, IL-15, and IL-18 become long-lived, memory-like (ML) NK cells with the ability to respond robustly upon restimulation with cytokines and activating receptors, including CD16 engagement with tumor-targeting mAbs (24, 39–41). Correlative immunology from a study using ML NK cells as a cellular therapy for relapsed refractory acute myeloid leukemia (AML) identified a negative association between CD8α expression on donor ML NK cells and treatment outcome, such that expression of CD8α was higher on donor NK cells in patients experiencing treatment failure (24). Further work identified that CD8α^–^ ML NK cells had enhanced proliferation in patients and that sorted CD8α^–^ ML NK cells had a proliferative advantage in vitro. However, the biology and mechanisms associated with this finding, and how they extend to conventional NK (cNK) cells, remain unclear.

Here, we examined the biology of CD8α in human cNK cells and discovered an unexpected time-dependent association with IL-15 signaling, metabolism, and proliferation. Furthermore, we define a functional role for CD8α in regulating human NK cell activation.

## Results

### CD8α^–^ NK cells have enhanced tumor control in vivo.

**Figure 1:**
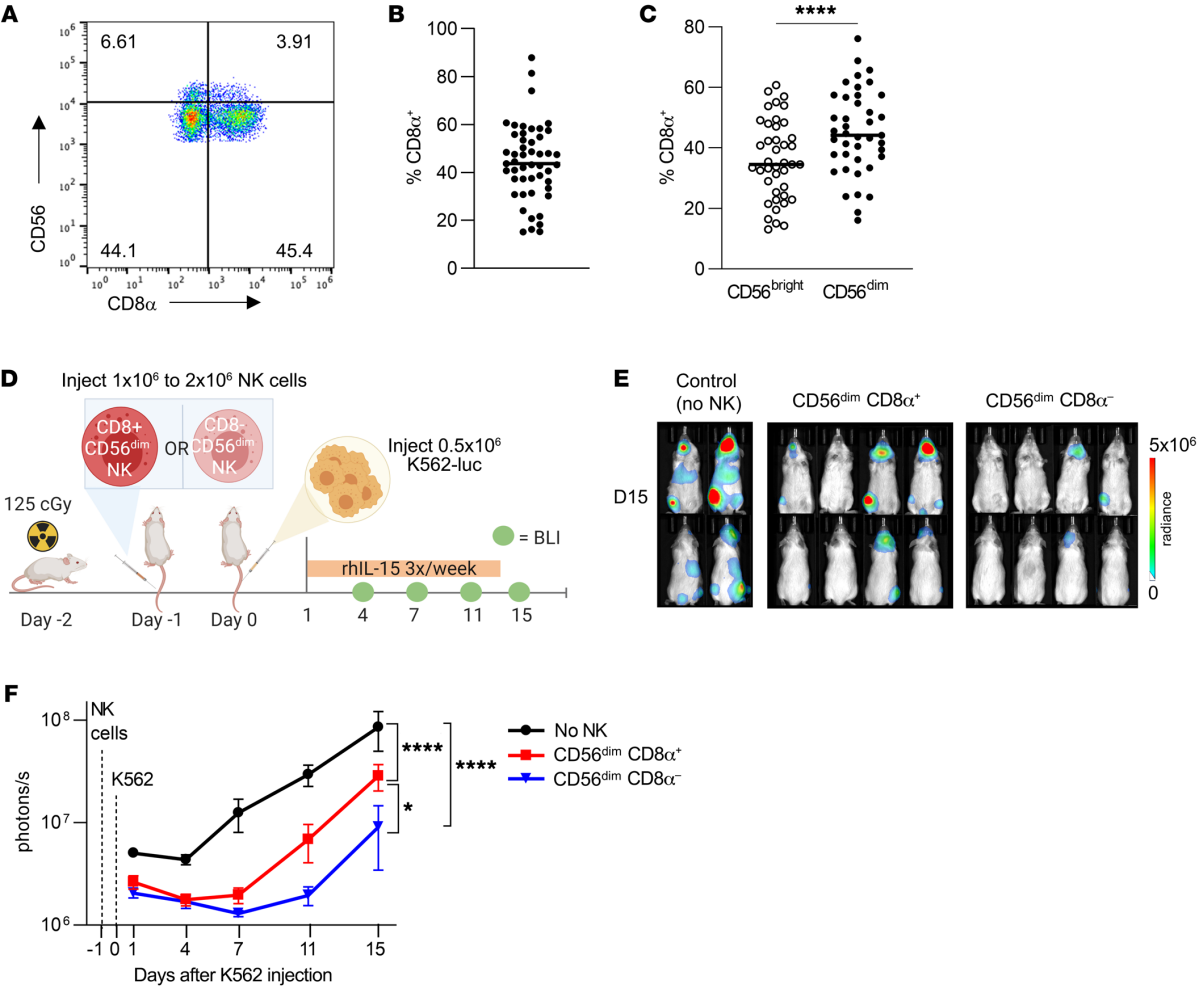
Sorted CD8α^–^ NK cells have enhanced tumor control in vivo. (**A**) Representative flow plot showing CD8α expression on CD56^bright^ and CD56^dim^ NK cells. (**B**). Percentage of freshly isolated healthy donor human NK cells that expressed CD8α. (**C**) Percentage of freshly isolated NK cells, gated into CD56^bright^ or CD56^dim^ cells, that expressed CD8α. *n* = 49. ****P < 0.0001, by 2-tailed, paired Student’s *t* test. (**D**–**F**) CD56^dim^CD8α^+^ and CD56^dim^CD8α**^–^** NK cells were sorted from primary human NK cells and rested overnight in 1 ng/mL IL-15. The next day, approximately 1 × 10^6^ to 2 × 10^6^ CD8α^+^ CD56^dim^ or CD8α**^–^**CD56^dim^ NK cells were injected i.v. via the tail vein into NSG mice (Day –1). The following day (Day 0), 0.4 × 10^6^ to 0.5 × 10^6^ K562-CBR-luciferase (K562-luc) cells were injected i.v. into the tail vein. NK cells were supported with i.p. rhIL-15 three times/week, and tumor burden was assessed via BLI on days 1, 4, 7, 11, and 15 after tumor injection. (**D**) Experimental schema. (**E**) Representative BLI images from 1 of 3 independent experiments on day 15 and (**F**) summary data showing tumor burden as the mean ± SEM within the indicated groups. *n* = 5 unique donors; *n* = 3 independent experiments; *n* = 8–10 mice in each group. *P < 0.05 and ****P < 0.0001, by mixed-effects model with Holm-Šídák correction for multiple comparisons.

Our prior study identified a negative association between CD8α expression on donor ML NK cells and treatment outcome and showed that ML CD8α^+^ NK cells have impaired proliferation in vitro (24). We examined the expression of CD8α on human cNK cells and found that CD8α was expressed by both CD56^bright^ and CD56^dim^ NK cell populations and that the percentage of CD8α^+^ NK cells was variable (Figure 1, A–C). Notably, a greater proportion of CD56^dim^ NK cells expressed CD8α compared with CD56^bright^ NK cells at baseline. Consistent with prior findings, CD8αα was the dominant form expressed, whereas a small fraction (<5%) of the cells expressed CD8αβ heterodimers (Supplemental Figure 1A; supplemental material available online with this article; https://doi.org/10.1172/JCI173602DS1) (24). In agreement with previous literature (23), we also confirmed that CD8α was not expressed on murine NK cells (Supplemental Figure 1B), precluding the findings from studies using murine models. We next sought to determine whether CD8α expression on the mature and cytotoxic CD56^dim^ cell population corresponded to differences in the ability to control tumors in vivo. CD8α^+^CD56^dim^ or CD8α^–^CD56^dim^ cNK cells were sorted from primary human NK cells and rested overnight in 1ng/mL IL-15. The next day (day –1), CD8α^+^CD56^dim^ or CD8α^–^CD56^dim^ cNK cells were injected i.v. into the tail vein of NOD-SCID-IL-2Rγ^–/–^ (NSG) mice, followed on day 0 with i.v. tail-vein injection of K562-CBR-luciferase cells. NK cells were supported with i.p. recombinant human IL-15 (rhIL-15) three times per week, tumor burden was measured via bioluminescence imaging (BLI) (Figure 1D). We found that mice treated with sorted CD8α^–^ NK cells had lower tumor burden compared with those that received CD8α^+^ NK cells or no NK cells at all (Figure 1, E and F). Notably, mice treated with CD8α^–^ or CD8α^+^ NK cells had similar tumor control initially (days 1 and 4), and differences between the groups became more apparent at later time points (days 7–15). Given that K562 cells lack HLA class I expression, we next sought to determine whether CD8α^–^ NK cells had enhanced responses against the HLA-expressing tumor cell lines Jeko-1 and HL60. Since NK cells are inhibited through KIR interaction with self-HLA, we compared the functional responses of CD8α^+^ or CD8α^–^ KIR3DL1, KIR2DL2/3, or KIR2DL1 single-positive (NKG2A^–^CD56^dim^) NK cells. We found that CD8α^–^ KIR2DL2/3 and KIR2DL1 single-positive NK cells had higher expression of IFN-γ following stimulation with both Jeko-1 and HL60 cell lines, compared with those that were CD8α^+^. There was no difference within KIR3DL1 single-positive NK cells, suggesting that this effect may have depended on the particular KIR-HLA combination engaged (Supplemental Figure 1, C–E). These data demonstrate that, compared with CD8α^+^ NK cells, CD8α^–^ NK cells had an enhanced capacity to control tumors in leukemia-xenografted mice and in vitro.

### CD8α^–^ NK cells have enhanced proliferation and survival in vitro and in vivo.

**Figure 2:**
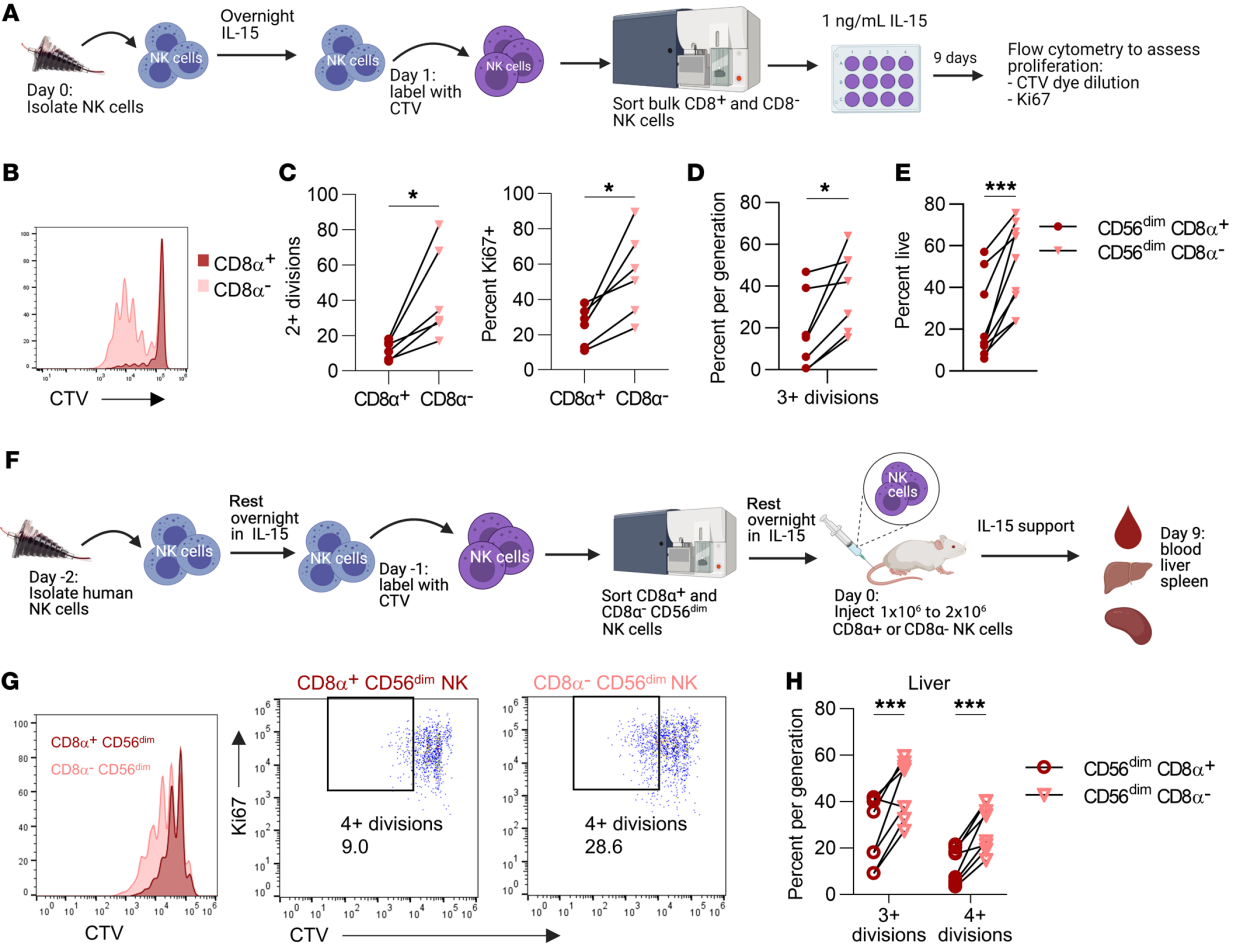
Sorted CD8α^–^ NK cells have enhanced proliferation and survival in vitro and in vivo. (**A**–**E**) Freshly isolated NK cells were labeled with CTV, sorted on the basis of CD8α expression, and cultured with 1 ng/mL IL-15 in vitro for 7 days. (**A**) Experimental schema. (**B**) Representative histogram of CTV dilution in CD8α^+^ and CD8α^–^ NK cells at day 7. Percentage of NK cells with (**C**) 2 or more divisions or Ki67 expression at day 7. *n* = 6 donors and 3 independent experiments. (**D**–**E**) CD8α^+^ or CD8α^–^ CD56^dim^ NK cells were labeled with CTV, sorted, and cultured in vitro in 1 ng/mL IL-15 for 9 days. (**D**) Proliferation was assessed by CTV dye dilution. Data are shown as the percentage of NK cells that had undergone the indicated number of divisions. (**E**) Cell death was assessed by staining with annexin V and 7AAD (live = annexin V^–^, 7AAD^–^). *n* = 7–9 donors and 4 independent experiments. (**F**–**H**) Sorted CD8α^+^CD56^dim^ and CD8α^–^CD56^dim^ NK cells were labeled with CTV and injected i.v. into different NSG mice. Human NK cells were supported with i.p. injections of rhIL-15 3 times/week. (**F**) Experimental schema. Proliferation was assessed by CTV dye dilution and Ki67 expression. (**G**) Representative histogram and (**H**) summary data showing the percentage of NK cells that had undergone the indicated number of divisions in the liver of NSG mice. Data represent the mean ± SEM. *P < 0.05 and ***P < 0.001, by (**C**–**E**) paired, 2-tailed Student’s *t* test and (**H**) 2-way ANOVA with Holm-Šídák correction for multiple comparisons. *n* = 9 donors and 5 independent experiments.

Since the ability of adoptively transferred allogeneic NK cells to eliminate residual leukemic cells relies on their persistence and expansion in vivo (40, 42), we sought to identify any proliferative differences between CD8α^+^ and CD8α^–^ NK cells. To determine the proliferative capacity of CD8α^+^ cNK cells, freshly isolated NK cells were labeled with CellTrace violet (CTV), sorted on the basis of CD8α expression, and cultured in IL-15 in vitro for 9 days (Figure 2A). Sorted CD8α^–^ NK cells exhibited significantly increased proliferation (Figure 2, B and C, and Supplemental Figure 2A) compared with sorted CD8α^+^ NK cells in vitro. Since CD56^dim^ NK cells had significantly higher expression of CD8α (Figure 1C) and are less proliferative than CD56^bright^ NK cells (1, 4), we also evaluated proliferation using sorted CD56^dim^ NK cells (Supplemental Figure 2B). Consistent with our observations with bulk NK cells, CD8α^–^CD56^dim^ NK cells were significantly more proliferative compared with sorted CD8α^+^CD56^dim^ NK cells (Figure 2D). Since IL-15 also regulates NK cell survival in addition to proliferation, we used 7AAD and annexin V staining to identify any differences in survival of these cell populations (14, 43, 44). Notably, CD8α^–^CD56^dim^ NK cells had significantly increased survival after IL-15 in vitro culturing compared with CD8α^+^CD56^dim^ NK cells (Figure 2E). On the basis of these in vitro studies, we hypothesized that CD8α^–^CD56^dim^ NK cells also have an enhanced proliferative capacity in vivo. Human cNK cells were labeled with CTV, CD56^dim^ NK cells were sorted on the basis of CD8α expression, and CD8α^+^CD56^dim^ or CD8α^–^CD56^dim^ NK cells were injected i.v. into NSG mice (Figure 2F). NK cells were supported with rhIL-15, and after 9 days, we isolated human NK cells from the blood, spleen, and liver of these mice. We found that sorted CD8α^–^CD56^dim^ NK cells had robust proliferation in the liver (Figure 2, G and H), in addition to the blood and spleen (Supplemental Figure 2, C and D). Furthermore, the absolute number of NK cells was significantly higher in the liver of mice that received CD8α^–^CD56^dim^ NK cells (Supplemental Figure 2E). Thus, sorted CD8α^–^CD56^dim^ NK cells underwent significantly increased proliferation and expansion in vitro and in vivo with IL-15 support compared with sorted CD8α^+^CD56^dim^ NK cells.

### CD8α does not mark a distinct, terminally differentiated cell population.

**Figure 3:**
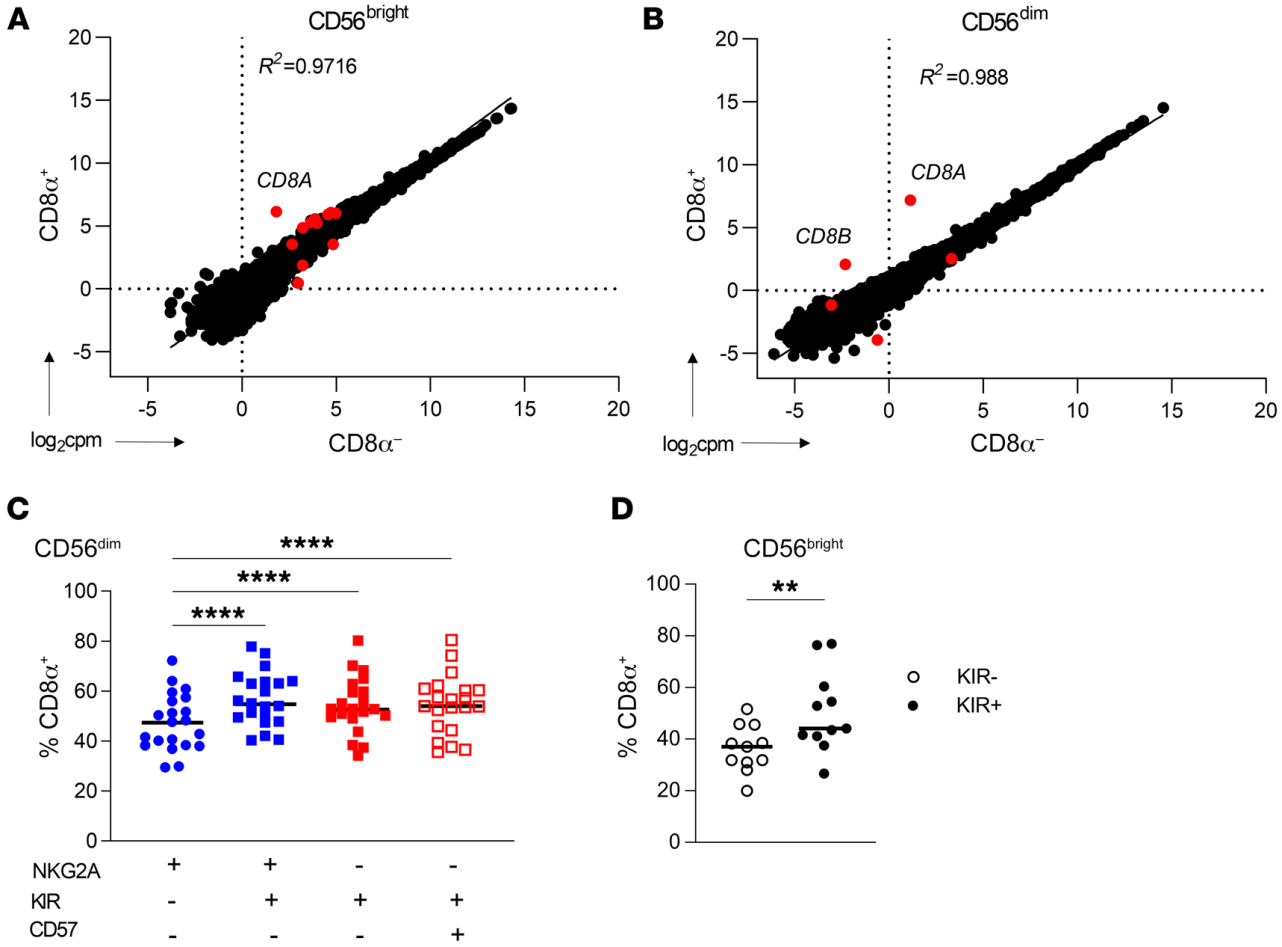
CD8α does not mark a distinct, terminally differentiated population. (**A** and **B**) Bulk RNA-Seq was performed on freshly isolated (**A**) CD56^bright^ or (**B**) CD56^dim^ NK cells sorted on the basis of CD8α expression (CD3^–^CD19^–^CD14^–^). Data are shown as the log_2_-normalized expression of protein-coding genes in CD8α^+/–^ cell populations. Red dots indicate genes that were statistically significantly differentially expressed (adjusted *P* < 0.05). *n* = 6 unique donors. The *R^2^* value was derived from simple linear regression of gene expression data. (**C** and **D**). Peripheral blood NK cells were stained for the expression of markers of NK maturation. (**C**) CD56^dim^ NK cell maturation stages were identified based on expression of NKG2A, KIR (KIR3DL1, KIR2DL1, and KIR2DL2/3), and CD57, with maturation increasing from left to right. Data are shown as the percentage of each subset that was positive for CD8α expression. *n* = 28 donors. (**D**) Expression of CD8α within NKG2A^–^CD56^bright^KIR^–^ or KIR^+^ (KIR3DL1^+^, KIR2DL1^+^, and KIR2DL2/3^+^) NK cells. *n* = 11 donors. Data represent the mean ± SEM. **P < 0.01 and ****P < 0.0001, by (**C**) 2-way ANOVA with Holm-Šídák correction for multiple comparisons and (**D**) paired, 2-tailed Student’s *t* test.

Next, we investigated potential mechanisms responsible for the proliferative differences based on CD8α expression. Since CD8α was expressed on both CD56^bright^ and CD56^dim^ NK cell subsets, we reasoned that, rather than marking a terminal differentiation event, CD8α may represent a distinct functional or activation state. This was first evaluated via bulk RNA-Seq of sorted CD8α^+/–^ CD56^bright^ and CD56^dim^ NK cells, which revealed similar transcriptional profiles between the populations (Figure 3, A and B, and Supplemental Figure 3, A and B). *CD8A* mRNA levels were markedly higher in CD8α^+^ NK cells, supporting the idea that transcript abundance was responsible for the CD8α protein differences. In addition, we analyzed single-cell RNA-Seq (scRNA-Seq) of primary human NK cells and found that CD8α did not associate with or identify a unique cell subset, as defined by uniform manifold approximation and projection (UMAP) clustering (Supplemental Figure 3, C and D). These data suggest that CD8α did not mark a subset of NK cells with a distinct gene expression program. Consistent with this finding, mass cytometry phenotyping of CD56^bright^ and CD56^dim^ cNK cells identified minor differences in the frequency of activating receptors (Supplemental Figure 3, E and F). To determine whether CD8α is associated with a particular maturation stage, we evaluated CD8α expression within an established progression of CD56^dim^ maturation, characterized by loss of NKG2A expression and acquisition of KIR (defined here as KIR3DL1^+^, KIR2DL1^+^, and KIR2DL2/3^+^) and CD57. CD8α expression was modestly increased on the terminally matured NKG2A^–^KIR^+^CD57^+^CD56^dim^ subset compared with the immature NKG2A^–^KIR^–^CD57^–^ population. Interestingly, CD8α expression was highest on KIR^+^CD56^dim^ and CD56^bright^ subsets compared with the KIR^–^ subsets (Figure 3, C and D). NK cells acquire functional competence via education or licensing through KIR interactions with self-HLA (45). Since CD8α binds HLA on the conserved α3 domain, while KIR binds a polymorphic site on the α2 domain, we hypothesized that CD8α may be enriched on KIR-licensed versus unlicensed NK cells. To assess this, we identified NKG2A^–^ KIR single-positive licensed or unlicensed CD56^dim^ NK cells (46) and compared licensed with unlicensed KIR^+^ NK cells within donors (Supplemental Figure 4A) or across individual KIR single-positive CD56^dim^ NK cells (Supplemental Figure 4B). This revealed no significant differences in CD8α expression based on self-KIR licensing status. Collectively, these data suggest that CD8α does not identify a distinct subset of NK cells defined transcriptionally, via maturation, or by licensing; however, CD8α expression was enriched on KIR^+^ NK cells.

### IL-15 modulates CD8α expression on CD8α^–^ NK cells via RUNX3.

**Figure 4:**
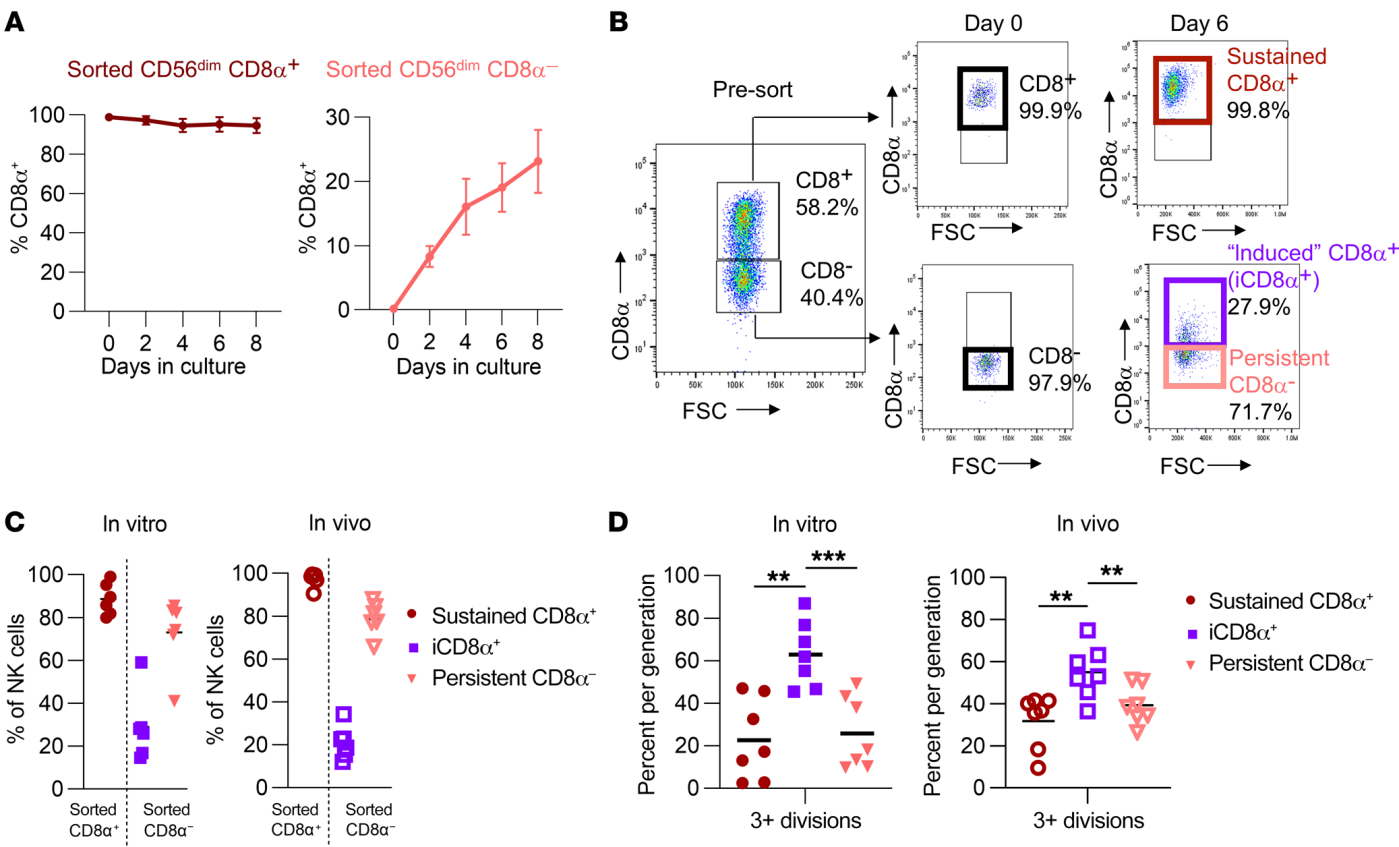
IL-15 modulates CD8α expression. (**A**) CD8α^+/–^CD56^dim^ NK cells were sorted and cultured in 5 ng/mL IL-15 for up to 8 days. Plots show the percentage of NK cells positive for CD8α expression on cells originally sorted as CD8α^+^ or CD8α^–^ cells. *n* = 2–3 donors and 2 independent experiments. (**B**) Gating strategy for identification of induced CD8α^+^ versus sustained CD8α^+^ and persistent CD8α^–^ NK cells. Sorted CD8α^+^ NK cells that remained CD8α^+^ were defined as sustained CD8α^+^ cells. Sorted CD8α^–^ NK cells that upregulated CD8α during culturing were defined as induced CD8α^+^ cells. Sorted CD8α^–^ NK cells that remained CD8α^–^ during culturing were defined as persistent CD8α^–^ cells. FSC, forward scatter. (**C** and **D**) CD8α^+/–^CD56^dim^ NK cells were sorted and cultured in 1 ng/mL IL-15 in vitro or injected into NSG mice supported with i.p. rhIL-15 3 times/week. Data are shown as the percentage of NK cells positive for CD8α expression after 9 days. *n* = 8 donors and 4 independent experiments. (**D**) Percentage of NK cells that underwent 3 or more divisions within the indicated subsets in vitro or in vivo in NSG mice 9 days after sorting. *n* = 6–9 donors and 4 independent experiments. Data represent the mean ± SEM. **P < 0.01 and ***P < 0.001, by 2-way ANOVA with Holm-Šídák correction for multiple comparisons.

**Figure 5:**
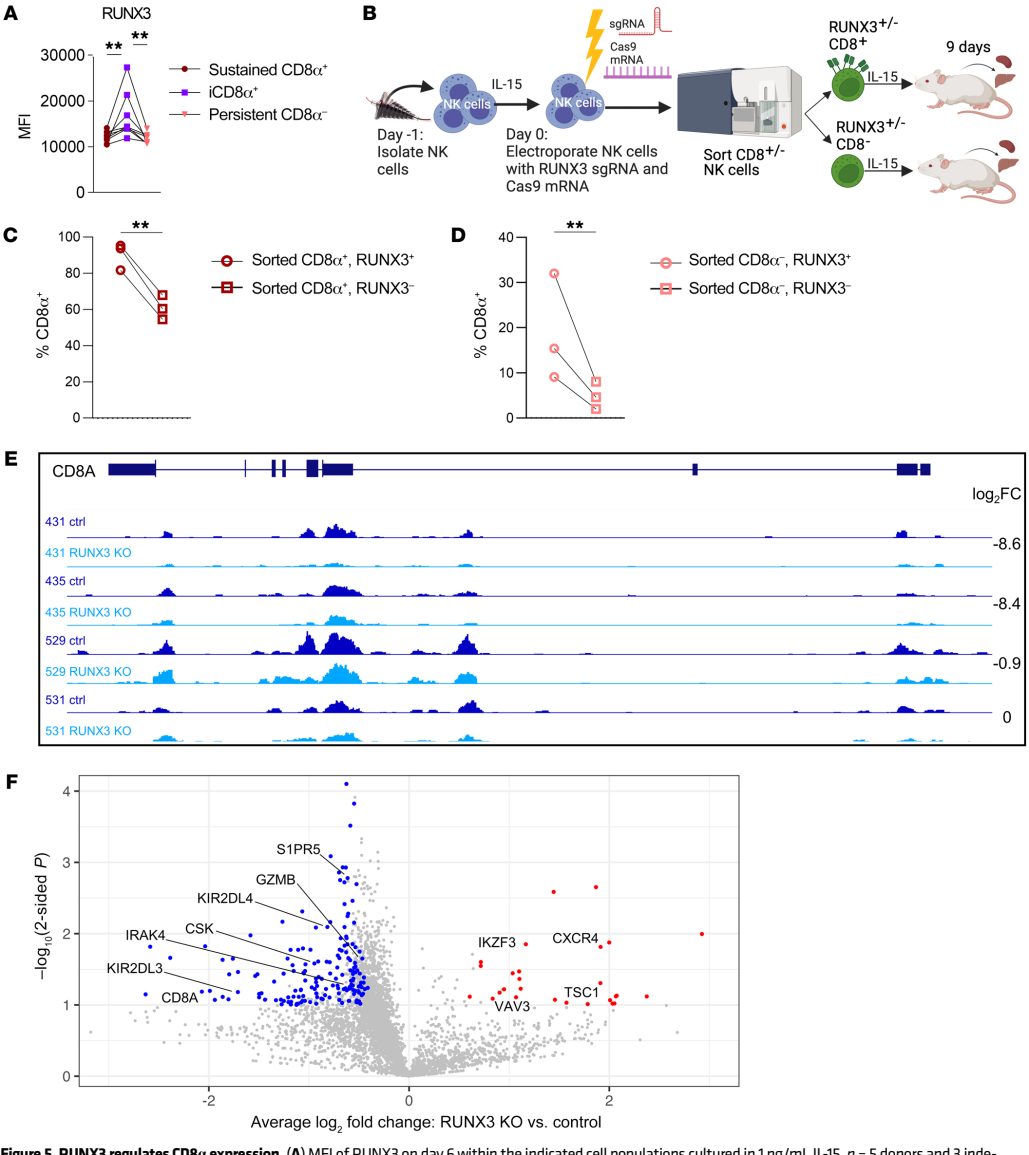
RUNX3 regulates CD8α expression. (**A**) MFI of RUNX3 on day 6 within the indicated cell populations cultured in 1 ng/mL IL-15. *n* = 5 donors and 3 independent experiments. (**B**–**D**) NK cells were electroporated with RUNX3 sgRNA and Cas9 mRNA, cultured in vitro for 48 hours, and then sorted on the basis of CD8α expression. NSG mice were injected i.v. with sorted CD8α^+/–^ control or RUNX3-KO cells and supported with i.p. rhIL-15 for 9 days. (**B**) Experimental schema. (**C** and **D**) Percentage of human NK cells in the liver expressing CD8α within RUNX3^+^ or RUNX3^–^ cell populations that were originally sorted as (**C**) CD8α^+^ or (**D**) CD8α^–^. *n* = 3 donors and 2 independent experiments. Data represent the mean ± SEM. **P < 0.01, by (**A**) repeated-measures 1-way ANOVA and (**C** and **D**) ratio-paired, 2-tailed Student’s *t* test. (**E** and **F**) NK cells were electroporated with control or RUNX3 gRNA and Cas9 mRNA, cultured in 5 ng/mL IL-15 for 9 days, and assessed for H3K27ac abundance using CUT&TAG. (**E**) Integrative Genomics Viewer (IGV) tracks showing H3K27ac peaks within the *CD8A* locus for control (ctrl) and RUNX3-KO donor pairs, with the log_2_ FC for each donor pair for the entire CD8A locus shown. (**F**) Volcano plot showing the average log_2_ FC and –log_10_ *P* value, determined by matched, paired, 2-tailed Student’s *t* test, for donor-matched RUNX3-KO versus control H3K27ac signal for gene loci. Genes in highlighted in red had significantly increased H3K27ac signal, and genes in blue had significantly decreased H3K27ac signal in RUNX3-KO cells with log_2_ FC cutoffs of absolute (0.5) or higher for at least 3 of 4 donors. We filtered genes with *P* < 0.05 using the results of 1-sided Student’s *t* tests (peaks lost/lower in KO or peaks gained/higher in KO), a log_2_ fold change ≤ -0.5 or ≥ 0.5, respectively) in at least 3 of 4 donors, for genes expressed in NK cells. *n* = 4 donors and 2 independent experiments.

CD8α is variably expressed on freshly isolated NK cells, and the signals that control expression are not defined. To address this, we sorted CD8α^+^ and CD8α^–^CD56^dim^ NK cells and evaluated CD8α expression after cytokine stimulation. We discovered that, while sorted CD8α^+^CD56^dim^ NK cells maintained CD8α expression, a subset of sorted CD8α^–^CD56^dim^ NK cells upregulated CD8α over time in culture (Figure 4A), and this effect was IL-15 dose dependent (Supplemental Figure 5A). In contrast, although CD56^bright^ NK cells had lower expression of CD8α on freshly isolated NK cells (Figure 1C), nearly all (80%) sorted CD8α^–^CD56^bright^ NK cells became CD8α^+^ in culture with IL-15 (Supplemental Figure 5B). The small percentage of CD8αβ^+^CD56^dim^ NK cells remained constant throughout stimulation, indicating that IL-15 independently induced CD8α, but not CD8β, expression (Supplemental Figure 5, C and D). This led us to hypothesize that the timing of CD8α acquisition may be a determinant of cytokine stimulation or proliferation status and that induced CD8α expression was marking a cell population that was robustly responding to IL-15 signals. To address this, CD8α^–^ CD56^dim^ and CD8α^+^ CD56^dim^ NK cells were sorted, cultured in vitro with IL-15 for at least 6 days, and gated according to CD8α expression (Figure 4, B and C). This approach allowed us to isolate the expression of CD8α in time and assess the biological features of “sustained” CD8α^+^ NK cells (sorted CD8α^+^ NK that sustained CD8α expression) versus “induced” CD8α^+^ (iCD8α^+^, sorted CD8α^–^ NK cells that acquired CD8α expression during culture) and persistent CD8α^–^ (CD8α^–^ NK cells that remained CD8α^–^). After 6 days, almost all sorted CD8α^+^ NK cells remained sustained CD8α^+^. However, approximately 30% of sorted CD8α^–^ NK cells became iCD8α^+^, and the remainder were persistent CD8α^–^ (Figure 4C). Unexpectedly, we discovered that NK cells with induced CD8α expression were the most proliferative, compared with those that remained CD8α^–^ or those that sustained CD8α expression, and this effect was consistent both in vitro and in vivo within NSG mice (Figure 4D). To further characterize the factors that regulate CD8α expression, we examined the expression of RUNX3, a transcription factor that has predicted binding sites located within putative regulatory regions in the *CD8A* gene locus (47). We found that RUNX3 expression was higher in iCD8α^+^ NK cells as compared with sustained CD8α^+^ or persistent CD8α^–^ NK cells (Figure 5A). Furthermore, we found that CRISPR/Cas9 gene editing of *RUNX3* and subsequent transfer into NSG mice to allow for robust proliferation (Figure 5B and Supplemental Figure 5E) resulted in decreased expression of CD8α in sorted CD8α^+^ NK cells (Figure 5C) and abrogated the upregulation of CD8α in sorted CD8α^–^ NK cells (Figure 5D). To further assess whether RUNX3 regulates CD8α at the transcriptional level, we used CUT&TAG (48) to compare the abundance of H3K27ac, an epigenetic modification of histones in promoters, enhancers, and gene bodies that is correlated with active transcription, in control and *RUNX3*-KO NK cells. Using log_2_ fold change (FC) and a matched, paired Student’s *t* test for RUNX3 deletion and control, we required a *P* value of less than 0.05 and that at least 3 of 4 donors had a log_2_ FC of absolute 0.5 or greater. We found that loss of RUNX3 led to a decrease in total H3K27ac signal within the *CD8A* locus with a log_2_ FC of –0.9 to –8.6 for 3 of 4 donors, with 1 donor having low H3K27ac abundance in both control and *RUNX3*-KO conditions (Figure 5E). This analysis also identified 174 genes with lower H3K27ac signal and 23 genes with higher H3K27ac signal in *RUNX3* KO NK cells. Notably, deletion of RUNX3 led to a decrease in H3K27ac peaks near genes involved in NK cell function and activation (*GZMB*, *CSK*, *LAT*, *IRAK4, TGFBR1*, *KIR2DL4*, *KIR2DL3*, *TNFSF14*, *PLCB2*, *CCL5*, *S1PR5*), translation initiation (*EIF2AK1*, *EIF2B2*), and nutrient transport (*SLC39A6*, *SLC1A5*, *SLC35A5*, *SLC50A1*), and an increase in H3K27ac peaks near genes related to NK cell development (*IKZF3*, also known as *Aiolos*) and signaling (*CXC4*, *TSC1*, *VAV3*); no H3K27ac peaks were detected in the *CD8B* promoter or gene body (Figure 5F and Supplemental Figure 5F). Taken together, these data demonstrate that IL-15 induces CD8α expression, recent CD8α upregulation marks highly proliferative cells, and RUNX3 regulates expression of *CD8A* and other genes related to NK cell proliferation and activation.

### IL-15R density determines NK cell proliferation and upregulation of CD8α after IL-15 stimulation.

**Figure 6:**
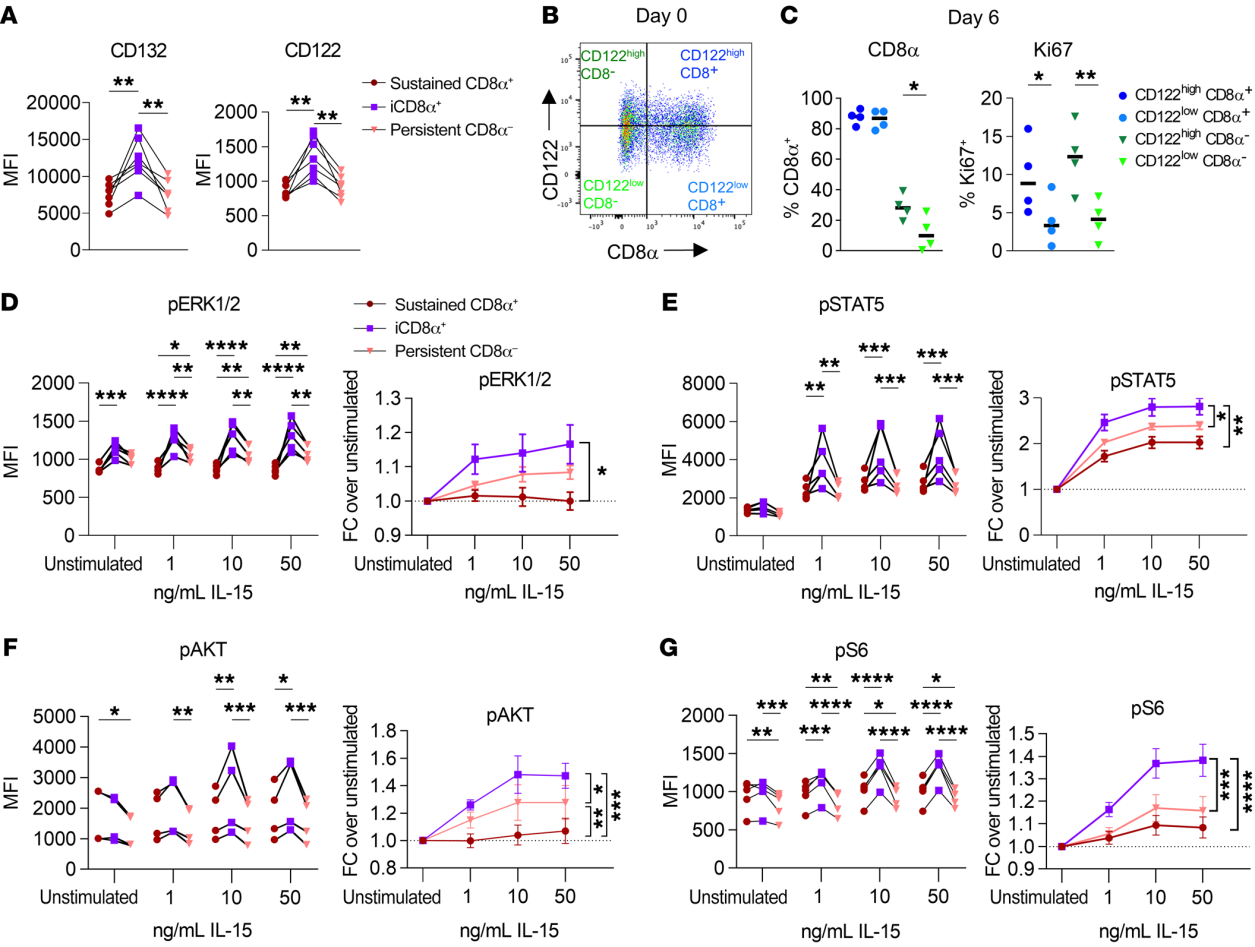
iCD8α NK cells have greater IL-15R expression and signaling. (**A**) Primary human NK cells were sorted into CD8α^+^CD56^dim^ and CD8α^–^CD56^dim^ populations and cultured in vitro in 1 ng/mL IL-15 for 6 days. CD132 and CD122 expression was assessed by flow cytometry, gated within the indicated subsets. *n* = 7 donors and 3 independent experiments. (**B** and **C**) CD56^dim^ NK cells were sorted from freshly isolated primary human NK cells, based on high and low expression of CD122 and CD8α, and cultured for 6 days in vitro in 5 ng/mL IL-15. (**B**) Representative flow plots of the gating strategy for cell sorting. (**C**) Summary data showing the percentage of NK cells positive for CD8α or Ki67 expression that were originally sorted as CD122^hi^ or CD122^lo^ and CD8α^+^ or CD8α^–^. *n* = 4 donors, and 2 independent experiments. (**D**–**G**) CD8α^+^ CD56^dim^ and CD8α^–^CD56^dim^ NK cells were sorted and cultured for 6 days in vitro with 1 ng/mL IL-15. Cells were cultured briefly (1 hour) in cytokine-free media prior to stimulation for 1 hour with the indicated concentrations of IL-15. Data are shown as the MFI and FC over the unstimulated condition within the indicated cell subsets for (**D**) p-ERK1/-2, (**E**), p-STAT5, (**F**) p-AKT, and (**G**) p-S6. *n* = 5 donors and 2 independent experiments. Data represent the mean ± SEM.*P < 0.05, **P < 0.01, ***P < 0.001, and ****P < 0.0001, by (**A** and **C**) repeated-measures, 1-way ANOVA and (**D**–**G**) 2-way ANOVA with Holm-Šídák correction for multiple comparisons.

There are three IL-15 receptor subunits: IL-15Rα, IL-2/15Rβ (CD122), and the shared common γ chain (γc, CD132) (14, 49). IL15Rα binds to IL-15 with high affinity and facilitates trans-presentation to the signaling components of the IL-15 receptor on NK cells (IL-15Rβγ_c_). While all mature NK cells are positive for IL-15Rβγ_c_ expression and begin to acquire and maintain CD122 (IL-2/15Rβ) as they progress to the CD56^bright^ stage (50), the receptor components are expressed at varying densities on human NK cells (17, 51). We hypothesized that differential expression of these receptor components could be driving the enhanced proliferation in iCD8α^+^ NK cells. Notably, we found that iCD8α^+^ CD56^dim^ NK cells had significantly higher expression of CD132 (γ_c_) and CD122 (IL-2/IL-15Rβ) (Figure 6A), while the differences were more modest in CD56^bright^ NK cells (Supplemental Figure 6A). We next asked whether iCD8α^+^ NK cells have greater upregulation of IL-15R components in response to IL-15, or whether existing heterogeneity in IL-15R expression leads to the upregulation of CD8α in cells with higher expression of the IL-15R. Indeed, we found that sorted CD8α^–^ NK cells originating from a high CD122 density had greater proliferation and upregulation of CD8α, as opposed to those originating from a CD122^lo^ group (Figure 6, B and C). This indicates that NK cells with higher expression of IL-15R components preferentially upregulate CD8α and expand in the presence of IL-15. IL-15 is the main driver of NK cell proliferation via signaling through 3 pathways: JAK1,-3/STAT5, Ras/Raf/Mek/Erk (MAPK), and PI3K/AKT/mTOR. In support of this, NK cells with higher expression of CD122/IL-15Rβ had higher resting and IL-15–induced levels of phosphorylated ERK (p-ERK) and p-S6 (Supplemental Figure 6B), while there were no significant differences in total protein levels of STAT5, ERK, AKT, or S6 (Supplemental Figure 6C). Thus, increased expression of IL-15R expression corresponded to enhanced responses to IL-15 signaling. As such, we hypothesized that the enhanced expression of IL-15R components in iCD8α^+^ NK cells could lead to distinct responses to IL-15 signals that could be driving the observed proliferation differences. To identify signaling differences, NK cells were briefly cytokine starved prior to stimulation with various concentrations of IL-15 for 1 hour, and phosphorylation of downstream mediators of IL-15 signaling was determined by intracellular flow cytometry. In unsorted, freshly isolated CD56^bright^ NK cells gated according to CD8α expression, there were modest differences in the induction of p-STAT5, p-ERK1/2, and p-AKT, whereas the induction of p-S6 was significantly higher in CD8α^–^CD56^bright^ NK cells (Supplemental Figure 6D). Within unsorted CD56^dim^ NK cells, we detected a modest but consistently greater induction of p-ERK1/-2, p-AKT, and p-S6 in CD8α^–^CD56^dim^ NK cells (Supplemental Figure 6E). However, when controlling for the timing of CD8α acquisition using sorted CD8α^–^CD56^dim^ NK cells, we identified higher p-ERK1/-2, p-STAT5, p-AKT, and p-S6 levels (by MFI and FC) in iCD8α^+^ NK cells compared with sustained CD8α^+^ or persistent CD8α^–^CD56^dim^ NK cells (Figure 6, D–G). Interestingly, within sorted CD8α^–^CD56^bright^ NK cells, both iCD8α^+^ and persistent CD8α^–^ subsets had greater induction of p-STAT5, p-AKT, and p-S6 compared with sustained CD8α^+^CD56^bright^ NK cells (Supplemental Figure 7, A–D). These data suggest that the temporal dynamics of IL-15–driven expansion of IL-15R^hi^ NK cells and enhanced IL-15 signals, marked by subsequent upregulation of CD8α, are a key determinant of proliferative capacity.

### Induced CD8α expression is associated with metabolic activity in NK cells.

**Figure 7:**
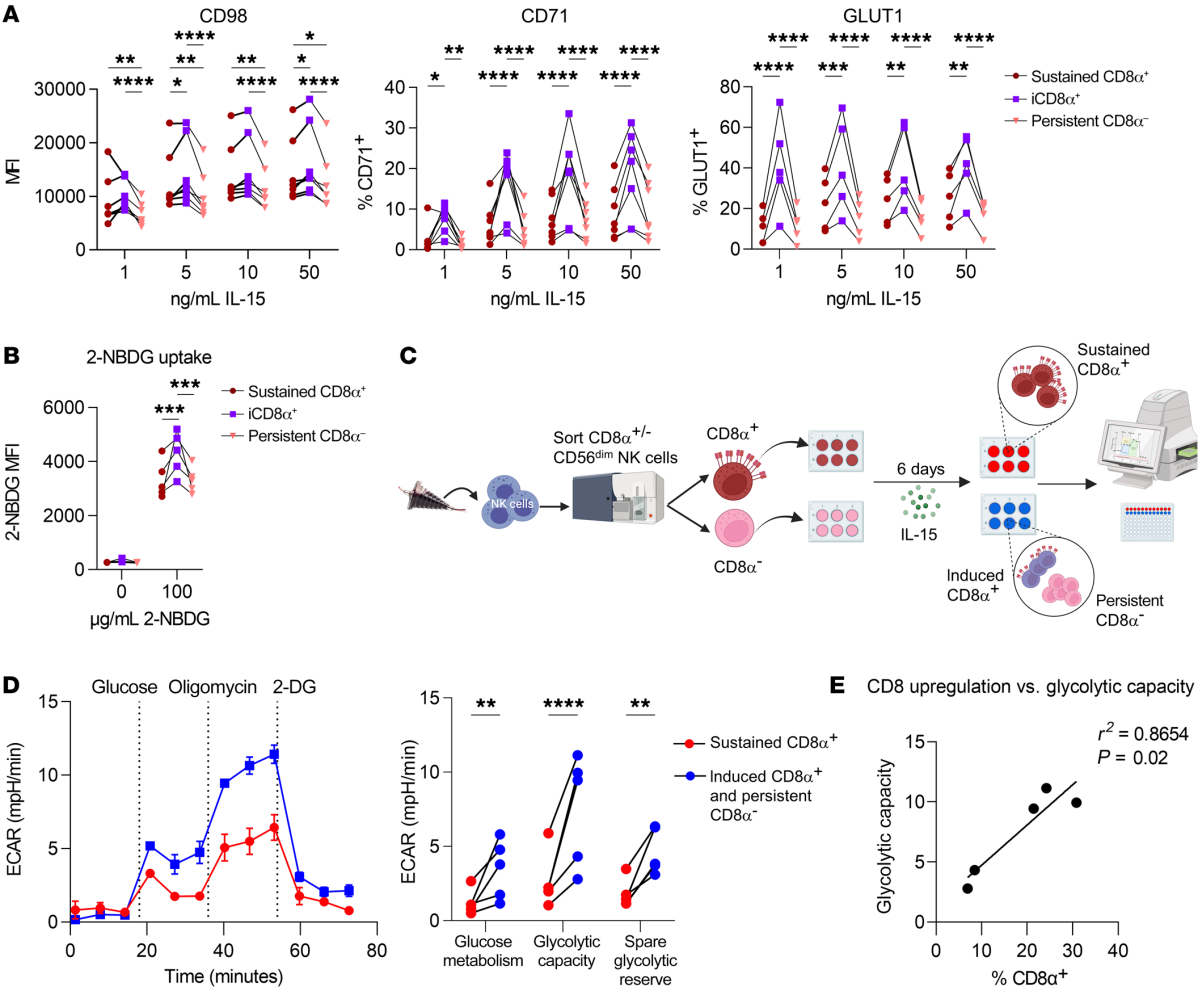
Induced CD8α expression is associated with metabolic activity in NK cells. (**A**) Primary human NK cells were sorted into CD8α^+^CD56^dim^ and CD8α^–^CD56^dim^ populations and cultured for 6 days in vitro with the indicated concentrations of IL-15. The MFI and percentage of NK cells positive for nutrient receptors CD98, CD71, and GLUT1 are shown. *n* = 7 donors, 3 independent experiments. (**B**–**E**) CD8α^+^ and CD8α^–^ NK cells were sorted and cultured for 6 days in vitro with 1 ng/mL IL-15. (**B**) Uptake of the fluorescent glucose analog 2-NBDG at various concentrations was assessed by flow cytometry. The MFI of 2-NBDG in the indicated subsets is shown. *n* = 7 donors and 3 independent experiments. (**C**–**E**) Metabolic parameters were determined using the Seahorse XFe96 Extracellular Flux Analyzer. (**C**) Experimental schema. (**D**) Donor glycolysis stress test trace from 1 representative donor, with measurement of the extracellular acidification rate (ECAR). The stimulation and summary data show glucose metabolism, glycolytic capacity, and glycolytic reserve. (**E**) Simple linear regression showing the relationship between the extent of CD8α upregulation within the sorted CD8α^–^ CD56^dim^ NK cells and the glycolytic capacity recorded via Seahorse. *n* = 6 donors and 4 independent experiments. Data represent the mean ± SEM. *P < 0.05, **P < 0.01, ***P < 0.001, and ****P < 0.0001, by 2-way ANOVA with Holm-Šídák correction for multiple comparisons.

The signaling pathways downstream from the IL-15R drive transcriptional and metabolic programs that control NK cell development, homeostasis, proliferation, and function (14, 19, 52). In particular, IL-15–induced mTOR activation is a key driver of NK cell proliferation via upregulation of nutrient receptors and proteins involved in glycolysis and lipid synthesis (19, 53). As such, we hypothesized that the observed differences in IL-15 signaling strength could translate to enhanced metabolic activity and drive the proliferation of iCD8α^+^ NK cells. We found that iCD8α^+^CD56^dim^ NK cells had dramatically higher expression of CD98 (amino acid receptor component), CD71 (transferrin receptor), and GLUT1 (glucose receptor) compared with expression levels in sustained CD8α^+^ or persistent CD8α^–^CD56^dim^ NK cells (Figure 7A). In CD56^bright^ NK cells, expression of CD71, but not CD98 or GLUT1, was higher in iCD8α^+^ NK cells, possibly because these proteins were highly expressed by nearly all CD56^bright^ NK cells following IL-15 culturing (Supplemental Figure 8, A–C). Consistent with the elevated expression of GLUT1 in CD56^dim^ NK cells, we identified greater uptake of the fluorescent glucose analog 2-NBDG in iCD8α^+^ NK cells, suggesting an enhanced capacity for glycolytic activity (Figure 7B). To further interrogate the metabolic activity of these cells, we performed Seahorse extracellular flux assays on sorted CD8α^–^ and CD8α^+^CD56^dim^ NK cells, with the caveat that these assays preclude the ability to differentiate iCD8α^+^ and persistent CD8α^–^ NK cells from the sorted CD8α^–^ group, although flow cytometric staining was performed at the conclusion of the assay to confirm CD8α induction in the CD8α^–^ group (Figure 7C). We found that sorted CD8α^–^CD56^dim^ NK cells had significantly higher glucose metabolism, glycolytic capacity, and spare glycolytic reserve compared with sorted CD8α^+^CD56^dim^ NK cells, suggesting a greater ability to engage in glucose-driven metabolic activity (Figure 7D). Additionally, we compared mitochondrial oxygen consumption rates (OCRs) and observed that sorted CD8α^–^CD56^dim^ NK cells had greater maximal respiration and spare respiratory capacity than did sorted CD8α^+^CD56^dim^ NK cells (Supplemental Figure 9A). The extent of CD8α upregulation in the sorted CD8α^–^CD56^dim^ group was positively correlated with a higher glycolytic capacity (Figure 7E) but not maximal respiration (Supplemental Figure 9B). These data suggest that the enhanced proliferative capacity previously identified in sorted CD8α^–^ NK cells was primarily driven by the iCD8α^+^ NK cells, which were more readily able to take up surrounding nutrients and upregulate the required glycolytic and oxidative machinery to engage in robust proliferation.

### Induction of CD8α corresponds to enhanced in vitro and ex vivo responses to tumors.

**Figure 8:**
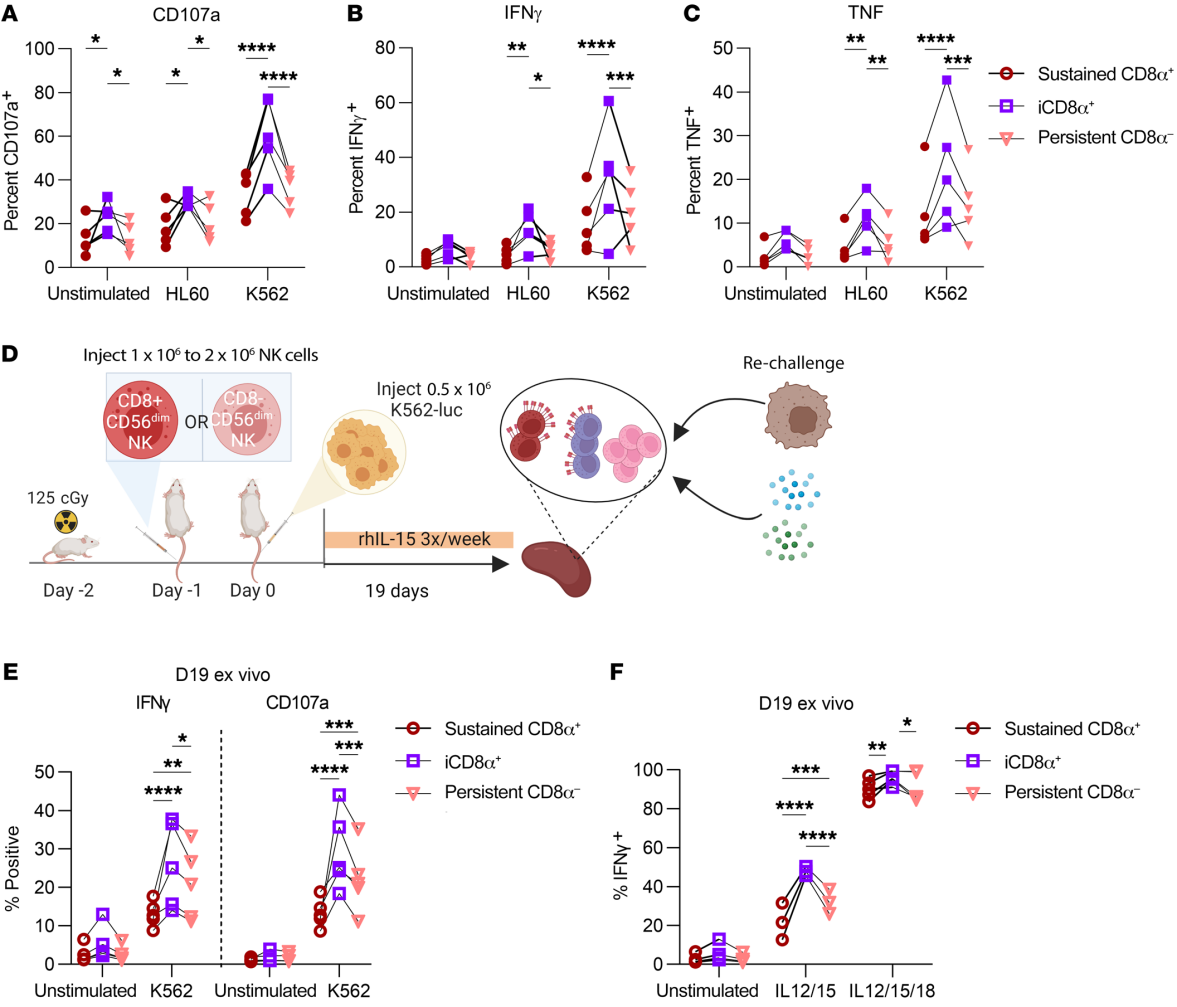
Induction of CD8α corresponds to enhanced in vitro and ex vivo responses to tumors. (**A**–**C**) Primary human NK cells were sorted into CD8α^+^CD56^dim^ and CD8α^–^CD56^dim^ populations and cultured in vitro in 5 ng/mL IL-15 for 6 days. NK cells were stimulated with HL60 or K562 leukemic cell lines at a 1:1 effector/target ratio for 6 hours, with GolgiPlug/Stop for the last 5 hours. Data are shown as the percentage of NK cells expressing (**A**) CD107a, (**B**) IFN-γ, or (**C**) TNF within the indicated cell subsets. *n* = 5 donors and 3 independent experiments. (**D**–**F**) CD56^dim^ NK cells were sorted on the basis of CD8α expression, and approximately 1 × 10^6^ to 2 × 10^6^ CD8α^+^CD56^dim^ or CD8α^–^CD56^dim^ NK cells were injected i.v. into the tail vein of NSG mice (day –1). The next day (day 0), 0.4 × 10^6^ to 0.5 × 10^6^ K562-CBR cells were injected i.v. into the tail vein. NK cells were supported with i.p. rhIL-15 three times/week. (**D**) Experimental schema. (**E** and **F**) On day 19, splenocytes were isolated from NK cell–treated mice and stimulated ex vivo with (**E**) K562s (10:1 splenocyte/K562 ratio) or (**F**) cytokines for 6 hours (20 ng/mL IL-12; 100 ng/mL IL-15; 100 ng/mL IL-18) with GolgiPlug/Stop in the last 5 hours. The percentage of NK cells positive for the indicated marker and gated within the indicated cell subsets is shown. *n* = 5 donors and 3 independent experiments. Data represent the mean ± SEM. *P < 0.05, **P < 0.01, ***P < 0.001, and ****P < 0.0001, by 2-way ANOVA with Holm-Šídák correction for multiple comparisons.

We next sought to determine whether this enhanced responsiveness to IL-15 signals and capacity for proliferation would translate to superior tumor control. Indeed, we found that iCD8α^+^ NK cells had greater activation, evidenced by surface CD107a and intracellular IFN-γ and TNF, following brief in vitro stimulation with K562 and HL60 leukemic cell lines (Figure 8, A–C). To determine whether iCD8α^+^ NK cells retained their enhanced functionality over longer time periods, we injected sorted CD8α^+^ and CD8α^–^ CD56^dim^ NK cells into NSG mice, infused them with K562 tumor cells the following day, and supported this with i.p. injections of IL-15 (Figure 8D). Surprisingly, even after almost 3 weeks of controlling tumor growth in vivo, iCD8α^+^ NK cells remained hyperfunctional to stimulation and had higher expression of IFN-γ and CD107a when rechallenged ex vivo with additional K562 cells or cytokines (Figure 8, E and F). This suggests that both the enhanced proliferation and cytotoxic function of iCD8α^+^ NK cells mechanistically contributed to the observed differences in tumor control (Figure 1) in this in vivo NSG mouse model.

### CD8α does not affect proliferation or apoptosis.

We next sought to determine whether CD8α itself could play a functional role in regulating proliferation or survival, as CD8α homodimers have been described in intraepithelial lymphocytes (54). In agreement with previous reports (31, 34), we observed that brief CD8α ligation with 2 mAb clones (RPA-T8 and SK1) induced intracellular calcium flux in a flow cytometry–based assay (Supplemental Figure 10A). Additionally, ligation of CD8α induced phosphorylation of PLCγ2, Lck, and S6, but not ZAP70/Syk, AKT, or ERK1/-2 (Supplemental Figure 10, B and C). This signaling induction was present in control, but not CD8 KO, cells (NK cells electroporated with CRISPR/Cas9 mRNA and gRNA targeting *CD8A*), confirming that this effect was not due to nonspecific antibody binding interactions. Despite the ability of CD8α to induce active signaling, we were unable to identify any effect of CD8 KO on survival or apoptosis in culture with IL-15. (Supplemental Figure 10, D–F). This indicated that CD8α does not intrinsically affect the ability of NK cells to expand in culture with IL-15.

### CD8α restricts NK-activating receptor function.

**Figure 9:**
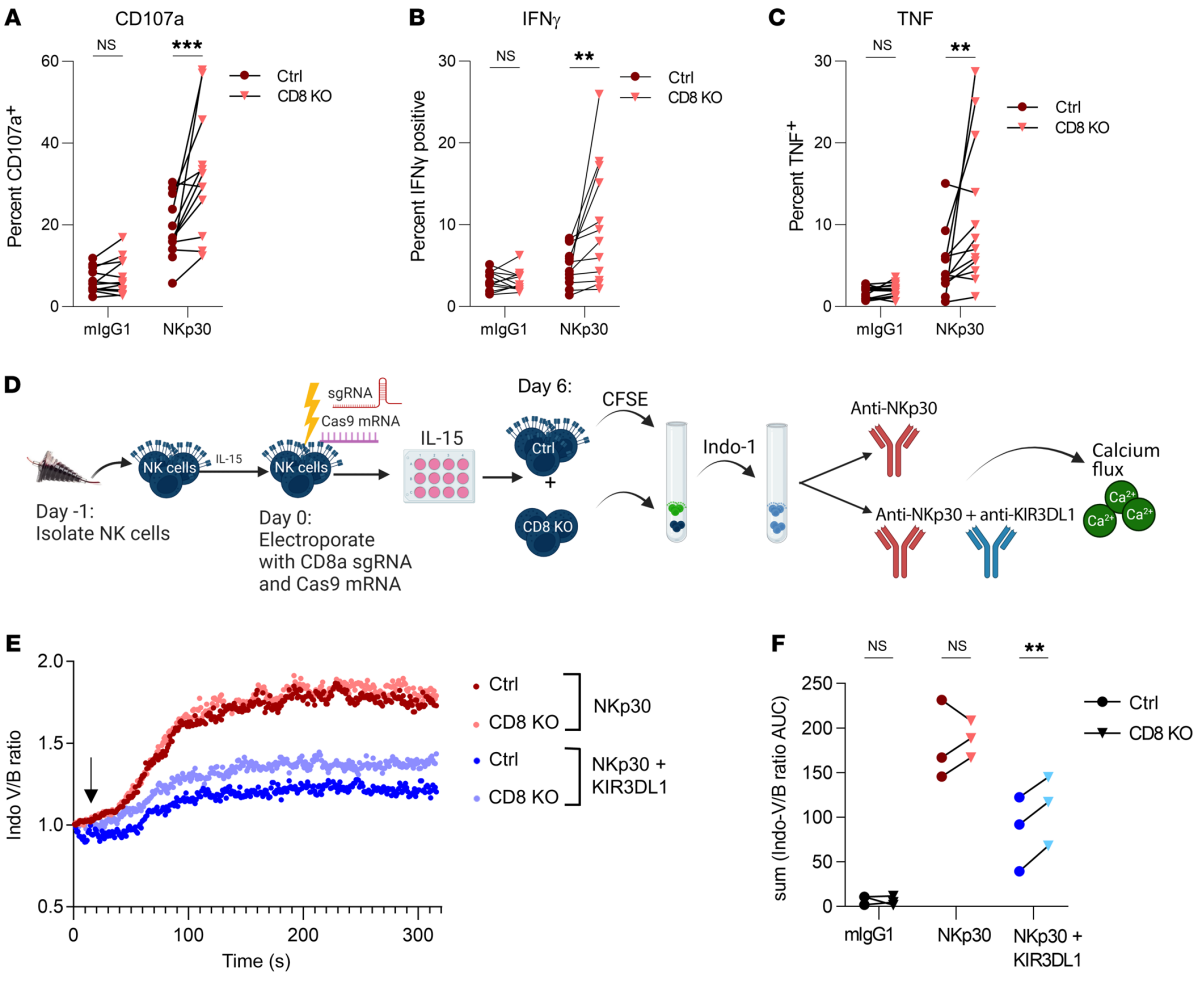
CD8A KO enhances cytokine secretion and degranulation following NKp30 stimulation. (**A**–**C**) Primary human NK cells were electroporated with Cas9 mRNA and sgRNA targeting *CD8A* or a control gRNA (*TRAC*) and cultured in vitro in 1 ng/mL IL-15 for 6 days. NK cells were stimulated with plate-bound antibodies (10 μg/mL) targeting NKp30 or mouse IgG1 isotype control antibody for 6 hours, with GolgiPlug/Stop for the last 5 hours. The percentage of NK cells positive for expression of (**A**) CD107a, (**B**) IFN-γ, or (**C**) TNF is shown. *n* = 13 donors and 7 independent experiments. (**D**–**F**) Control or CD8-KO cells were labeled with 50 nM CFSE, mixed together at a 1:1 ratio, and then labeled with the UV-excitable, Ca^2+^-sensing dye Indo-1. A mAb (5 μg/mL) targeting NKp30 alone or NKp30 (5 μg/mL) and KIR3DL1 (0.2 μg/mL) was added for 20 minutes at 4°C, cells were washed, and then cross-linking was induced at the indicated time point (black arrow) using goat anti–mouse IgG (10 μg/mL). Calcium flux was measured by flow cytometry. (**E**) Data are shown as the normalized ratio of Indo-violet over Indo-blue within control or CD8-KO cells as a function of time in cells from 1 representative donor. (**F**) Sum of the AUC of the normalized Indo-violet/Indo-blue ratio for all time points in control and CD8-KO cells. *n* = 3 donors and 3 independent experiments; donors were prescreened to ensure KIR3DL1 and CD8α expression of greater than 30%. Data represent the mean ± SEM. **P < 0.01 and ***P < 0.001, by (**A**–**C**) 2-way ANOVA with Holm-Šídák correction for multiple comparisons and (**F**–**G**) paired, 2-tailed Student’s *t* test.

CD8αα has been described on T cells to act as a TCR corepressor and on NK cells as a coreceptor that can enhance KIR clustering and binding to its cognate HLA-I ligand (26). Given that the cytoplasmic tail of CD8α can associate with Lck, an Src kinase that has been implicated in phosphorylation of both NK-activating and -inhibitory receptors (55), we next sought to determine whether CD8α instead played a role in modulating NK cell effector function. We found that CD8 KO had a minimal effect on responses to cytokines or HL60 cells, but led to modestly higher degranulation (CD107a) and TNF production against K562 leukemia cells (Supplemental Figure 11A). We also did not observe an effect on specific lysis of either K562 or HL60 tumor targets (Supplemental Figure 11, B and C) in a short-term (6-hour) killing assay. K562 cells are sensitive to NK cell killing because they lack HLA-class I expression and express multiple activating receptor ligands, so we hypothesized that CD8α could tune the activity of specific activating receptors. We briefly stimulated control or CD8-KO primary NK cells with plate-bound antibodies directed against activating receptors with shared (CD16, NKp30, NKp46) and distinct (CD2, CD226, 2B4, NKG2D) signaling adaptors and measured degranulation (CD107a) and cytokine production (IFN-γ, TNF). Notably, we found that CD8 KO led to significantly higher expression of IFN-γ, TNF, and CD107a following stimulation with NKp30 (Figure 9, A–C) and, to a lesser extent with 2B4, compared with control NK cells (Supplemental Figure 12, A–C). The effect on other activating receptors such as CD16 and CD2 was subject to greater donor-to-donor variability. Since CD8 KO had the greatest effect on NKp30 ligation, we focused on its interactions with this receptor. CD8α lacks a palmitoylation site that allows association with lipid rafts (56), and it has been proposed that CD8α could sequester Lck away from participating in proximal signaling events (26, 57). Successful activation of NK cells and target cell killing involves the recruitment and localization of activating receptors and the polarization of perforin-containing granules at an activating synapse (58). We therefore hypothesized that CD8α would be localized outside of these synapses. Unexpectedly, CD8α was not excluded from synapses formed against K562 or HL60 target cells and trended toward being enriched in these synaptic areas (Supplemental Figure 13, A and B). We were also unable to detect robust differences in the ability to signal through activating receptors, as measured by intracellular flow cytometric assessment of key phosphorylated signaling molecules (ZAP70, PLCγ2, S6, ERK1/2, Lck, AKT) (Supplemental Figure 14) following activating receptor ligation in control or CD8-KO cells. Interestingly, in CD56^dim^ NK cells, CD8 KO led to a modest increase in p-PLCγ2, p-Lck, p-ZAP70, and p-AKT following CD16 ligation compared with control NK cells, but we were unable to detect robust signaling following NKp30 ligation with this approach. Given the technical difficulties in capturing the kinetics of many signaling proteins at a single snapshot in time using phosphoflow, we used calcium flux to provide an integrated assessment of signaling. Since Lck has also been implicated in phosphorylating the immunoreceptor tyrosine-based inhibitory motifs (ITIMs) of KIR (55, 59, 60), and KIR and CD8α bind to nonoverlapping regions of HLA (61, 62), we hypothesized that instead of suppressing activating receptor function, CD8α could be enhancing the inhibitory function of KIR. Interestingly, we found that, while CD8 KO had no effect on calcium flux following NKp30 ligation alone, CD8 KO cells were less sensitive to KIR-mediated inhibition of NKp30, suggesting that the presence of CD8α facilitated KIR function (Figure 9, D–F). Together, these data suggest that CD8α can play an inhibitory role in NK cell function, probably due to its effects on KIR-mediated inhibitory signaling rather than direct modulation of activating receptor function.

## Discussion

Here, we show that sorted CD8α^–^CD56^dim^ NK cells had superior tumor control in vivo, likely due to enhanced IL-15–induced proliferation. This phenotype was not clearly linked to terminal maturation, as CD8α expression was highest on KIR^+^CD56^dim^ NK cells regardless of maturation or licensing status and there were few transcriptional differences between CD8α^+^ or CD8α^–^ cells within CD56^bright^ and CD56^dim^ NK cell subsets. Interestingly, we observed that CD8α expression was dynamic, and in the presence of IL-15, a subset of sorted CD8α- NK cells induced CD8α expression, while the majority remained CD8α^–^ and CD8α^+^ NK cells maintained CD8α expression once it had been established. Further analysis of the iCD8α^+^ subsets revealed that they drove the majority of the enhanced proliferation previously identified in sorted CD8α^–^CD56^dim^ NK cell subsets. Deletion of *CD8A* with CRISPR/Cas9 had no effect on proliferation or survival, suggesting that the intrinsic function of CD8α was unrelated to this enhanced IL-15–induced functionality. Mechanistically, iCD8α^+^ NK cells had higher expression of IL-15Rβ and γ receptor subunits, which resulted in significantly higher activation of the STAT5 and PI3K/AKT/mTOR pathways following IL-15 stimulation compared with sustained CD8α^+^ and persistent CD8α^–^ subsets. Interestingly, rather than IL-15–induced upregulation of IL-15R components, existing heterogeneity in IL-15R expression led to a preferential expansion and upregulation of CD8α in NK cells that have high expression of IL-15Rβ. These enhanced signals translated to greater glucose uptake and expression of nutrient receptors in iCD8α^+^ NK cells, suggesting that the iCD8α^+^ NK cells were responsible for the higher levels of glycolysis and oxidative phosphorylation seen in sorted CD8α^–^ NK cells.

IL-15–mediated upregulation of CD8α expression on NK cells was at least partially controlled by the transcription factor RUNX3, which has been implicated in IL-15–induced activation and proliferation of murine NK cells and in regulating CD8α expression in T cells (63, 64). We found that RUNX3 expression was higher on iCD8α^+^ NK cells in IL-15 culture and that RUNX3 KO via CRISPR/Cas9 resulted in the loss of CD8α expression in CD8α^+^ sorted cells and a restricted ability of CD8α^–^ NK cells to upregulate CD8α. This effect was specific to CD8α, and not CD8β. Furthermore, using CUT&TAG, we found that RUNX3 modulated H3K27ac abundance within the *CD8A* locus, indicating a direct role for RUNX3 in regulating CD8α expression. Notably, RUNX3 also regulated the expression of several KIR genes, in addition to genes related to NK cell activation and translation initiation/nutrient transport, suggesting that RUNX3 may facilitate the enhanced proliferation and effector function of induced CD8α^+^ NK cells. However, given that RUNX3 can form a heterodimer with CBFβ and has also been described to act in concert with T-box transcription factor 21 (TBET), further work should be done to determine the contribution of other transcription factors to CD8α expression (64, 65). Future studies interrogating other epigenetic modifications such as H3K4me3 and transcriptional activators such as Mediator may further elucidate the transcriptional program regulated by RUNX3 and clarify whether it directly or indirectly regulates CD8α and other IL-15–induced transcriptional programs.

In addition to the role of IL-15 in driving NK cell survival and proliferation, it has been demonstrated that IL-15–induced glucose metabolism is required for NK cell effector function (22). Consistent with this, the amino acid–sensing CD98/mTOR pathway has been shown to be critical for NK cell metabolism and effector function (66, 67). Our observation that iCD8α^+^ NK cells had greater upregulation of nutrient receptors suggests that they had improved metabolic support for enhanced proliferation and antitumor effector function. Consistent with this, we found that iCD8α^+^ NK cells had higher functionality against both the HLA-deficient K562 cell line and the HLA-sufficient HL60 cell line, suggesting that the presence or absence of HLA on a target cell did not necessarily affect this enhanced functionality. We also found that sorted CD8α^–^ NK cells had a superior ability to control tumors in vivo, likely owing to the enhanced proliferation and durable functionality of iCD8α^+^ NK cells, as opposed to those that were sorted as CD8α^+^ and had sustained CD8α expression.

Altogether, these data suggest that our previous observation that patients treated with ML NK cells with higher initial CD8α expression experienced treatment failure could have resulted from the hypofunctionality of sustained CD8α^+^ NK cells (24). Surprisingly, even after almost 3 weeks in vivo in the presence of K562 tumor cells and high doses of IL-15, iCD8α^+^ NK cells had enhanced responses to K562 and cytokine stimulation compared with persistent CD8α^–^ or sustained CD8α^+^ NK cells. This suggests that the loss of functionality associated with sustained CD8α^+^ expression occurred at time points that were far beyond the half-lives of adoptively transferred NK cells or was driven by additional tissue-specific factors not evaluated (68).

The CD8αα homodimer has been described to act as a TCR corepressor that can decrease TCR functional avidity, thus increasing the signal strength required for T cell activation (69, 70). Indeed, studies in intraepithelial lymphocytes (IELs) have demonstrated that CD8αα binding to thymus leukemic antigen (TL) restricts IEL proliferation and activation (71). Since the CD8α cytosolic tail binds the Src kinase Lck and lacks the palmitoylation site that allows close association with lipid rafts (56, 72, 73), Lck sequestration is proposed to be important for CD8αα inhibitory activity. Interestingly, Lck is also required for the phosphorylation of the ITIM domains of inhibitory KIRs that are clustered at the immune synapse (55, 60, 62), thus facilitating recruitment of the phosphatases SHP-1 and SHP-2 (74, 75). As such, it is also possible that CD8α binding to HLA class I in concert with KIR can modulate NK cell activity. It was reported that CD8αα can function as a coreceptor that enhances KIR clustering and binding to its cognate HLA-I ligand on adjacent NK cells, thereby increasing the inhibitory effect of KIRs (36). In this study, we found that CRISPR/Cas9 KO of *CD8A* in primary human NK cells led to enhanced NK cell degranulation and cytokine secretion following ligation with various activating receptors, particularly NKp30. Interestingly, we could not identify any direct effect of CD8α on activating coreceptor signaling, but rather a decreased sensitivity to KIR-mediated inhibition in the absence of CD8α. Notably, we found that CD8α expression was enriched on KIR^+^ NK cells. Therefore, we propose a model whereby IL-15 stimulation induces robust NK cell proliferation, metabolic activity, and cytotoxic functional capacity that is marked by CD8α expression, which subsequently acts as a rheostat to tune the threshold to release KIR-mediated inhibition and prevent aberrant activation. This finding is consistent with a potential role for CD8α as a coreceptor for KIR3DL1 on NK cells, although further work is required to define the exact mechanism by which CD8α functions and to determine whether this extends to other members of the KIR family. We hypothesize that after sufficient IL-15 signaling and time, iCD8α^+^ NK cells may transition to a phenotype that more closely resembles sustained CD8α^+^ NK cells. To that end, the impaired functionality of sustained CD8α^+^ NK cells may be 2-fold — as a consequence of an exhaustion-like state from chronic stimulation (manifesting as reduced responses to IL-15 signaling and lesser functional responses) and due to high expression of CD8α (tuning NK cell activation). In the context of responses against HLA-deficient K562s, the former effect may predominate, whereas against HLA-expressing cells it may be a combination of the two. Notably, the per-cell expression of CD8α (as measured by MFI) on iCD8α^+^ NK cells is much lower than that of sustained CD8α^+^ NK cells, suggesting that iCD8α^+^ NK cells are at least transiently less susceptible to inhibitory effects of CD8α. Finally, while K562s lack HLA expression, it is also possible that some level of tonic inhibition is tumor-target independent and is mediated by NK-NK interactions of CD8α/KIR-HLA.

In summary, this study reveals that CD8α expression on NK cells marked a spectrum of functionality, whereby recent induction of CD8α expression by CD8α^–^ NK cells corresponded with robust proliferation, metabolic activity, and functional responses. CD8α^–^ NK cells, which could remain CD8α^–^ or become iCD8α^+^, mediated superior tumor control in leukemia xenograft mouse models, likely due to their enhanced capacity for expansion in vivo. This enhanced functionality was lost over time, as sustained expression of CD8α was associated with hypofunctionality. Finally, this study identifies a functional, inhibitory role for CD8α in regulating NK cell function. These findings highlight the importance of interrogating the dynamics of NK cell marker acquisition as they relate to functionality, particularly in the context of understanding NK cell biology and improving cellular therapies.

## Methods

### Sex as a biological variable.

Experiments were performed on male and female mice that were age and sex matched within the experiments. No differences between sexes were observed.

Additional details on methods can be found in the Supplemental Methods.

### CRISPR/Cas9 gene editing.

NK cells from healthy donors were purified and rested overnight in HAB10 with 1 ng/mL IL15. Cells were washed twice with PBS to remove serum and resuspended in MaxCyte EP buffer plus Cas9 mRNA (Trilink). Next, CD8A sgRNA (GACUUCCGCCGAGAGAACGA) (IDT with modifications as described previously; ref. 76), *RUNX3* sgRNA (UGCGCACGAGCUCGCCUGCG), or control sgRNA against the TCRα chain TRAC (GAGAAUCAAAAUCGGUGAAU) (Synthego) was added to the cells, which were then electroporated in a Maxcyte GT electroporator using the WUSTL-2 setting in an OC-100 processing assembly. Cells were removed from the OC-100 and incubated for 10 minutes at 37°C. Prewarmed media containing 1 ng/mL IL15 were added, and cells were rested for 24 hours. Cells were then spun down (400*g*, 4 min) and resuspended at 2.5 × 10^6^ to 5 × 10^6^ cells/mL and cultured as described above. Protein KO was confirmed by flow cytometric staining at the indicated time points.

### Antibody cross-linking phosphorylation assays.

Six days after electroporation and culturing in HAB10 (RPMI 1640 supplemented with l-glutamine, HEPES, NEAA, sodium pyruvate, and penicillin/streptomycin/glutamine containing 10% FBS; Hyclone, GE Healthcare) plus 1 ng/mL IL-15, control or CD8-KO NK cells were plated at approximately 2.0 × 10^5^ to 2.5 × 10^5^ cells/well of a round-bottomed, 96-well plate in HAB10 containing 1 ng/mL rhIL-15. mAbs directed against the indicated activating receptors were added at 10 μg/mL, and incubated for 20 minutes at 4°C. Following incubation, cells were spun down at 750*g* for 4 minutes and resuspended in HAB10 containing 20 μg/mL goat anti–mouse IgG to induce cross-linking for 5 minutes at 37°C. After incubation, cells were fixed with 4% paraformaldehyde (PFA), permeabilized with methanol, and stained for flow cytometric analysis as described above.

### CUT&TAG data generation and analysis.

Freshly isolated NK cells were electroporated with RUNX3 or control (TRAC) sgRNA and Cas9 mRNA as described above, cultured in 5 ng/mL IL-15 for 9 days, and assessed for H3K27ac abundance using the Active Motif CUT&Tag-IT Anti-Rabbit assay kit. For each condition, 500,000 fresh, whole cells were used. RUNX3-KO efficiency of approximately 70%–80% was validated by flow cytometry on the day of sample preparation. The resulting libraries were submitted to the Washington University McDonnell Genome Institute for sequencing on an Illumina NovaSeq 6000 instrument. For analysis, CUT&TAG fastq files were aligned and analyzed using a pipeline adapted from refs. 77–79, using bowtie2, samtools, bedtools, and SEACR. H3K27ac bedgraph files for control and RUNX3 KO were compared with donor-matched IgG bedgraph files to identify H3K27ac peaks. Genome annotation of peaks was performed with ChIPseeker using promoter = ±3 kb and assigning the nearest gene to each peak; 9,558 genes had peaks assigned. Statistical analyses, filtering, and data visualization were performed using R. The total signal in peaks assigned to genes was compared between control and KO conditions for each donor using matched, paired Student’s *t* tests and log_2_ FC. We filtered genes with a *P* value of less than 0.05 using the results of 1-sided Student’s *t* tests (peaks lost/lower in KO or peaks gained/higher in KO; log_2_ FC ≤–0.5 or ≥0.5, respectively) in at least 3 of 4 donors for genes expressed in NK cells. This strategy identified 174 genes with lost or lower peaks in KO and 23 genes with gained or higher peaks in KO. Data are uploaded in the NCBI’s Gene Expression Omnibus (GEO) database under accession number GSE263686.

### Calcium flux assays.

Calcium flux assays were performed by washing freshly isolated human NK cells with prewarmed MCF media (HBSS with calcium [Thermo Fisher Scientific], 1% 1M HEPES [Thermo Fisher Scientific], and 2% human serum) and incubating the NK cells with Indo-1, AM (Thermo Fisher Scientific), a UV light–excitable Ca^2+^ indicator (emission maximum of Indo-1 shifts from ~475 nM in Ca^2+^-free medium to ~400 nM when the dye is saturated with Ca^2+^) for 30 minutes at 37°C, with mixing performed every 10 minutes. Indo-1–labeled cells were washed, resuspended with 10 μg/mL mAb or mIgG1 isotype control and incubated at 37°C for 15 minutes. Cells were washed and resuspended in MCF media and rested for 37°C for at least 15 minutes prior to acquisition on a UV laser–equipped FACSAria II on a flow rate of approximately 2,000 events/second for 15 seconds. Acquisition was paused, 20 μg/mL goat anti–mouse IgG was added to induce cross-linking, cells were vortexed, and acquisition was resumed for 5 minutes. Data analysis was performed by comparing the ratio of the median fluorescence intensity of Indo-blue and Indo-violet over the time series and normalized to the first reading using FlowJo, version 10.8.1 software (TreeStar).

### NSG xenograft model and BLI imaging.

Approximately 1 × 10^6^ to 2 × 10^6^ CD8α^+^ or CD8α^–^ CD56^dim^ NK cells were injected i.v. into the tail vein of NSG mice. The next day, 0.4 × 10^6^ to 0.5 × 10^6^ K562luciferase–expressing cells were injected i.v. into the tail vein. NK cells were supported with i.p. 1 μg/mouse rhIL-15 three times per week, and tumor measurements were assessed via BLI on days 1, 4, 7, 11, and 15 after tumor injection. All mice were irradiated with 125 cGy one day before NK cell injection. For each treatment condition, sorted NK cells were injected into 1–2 mice each, and the data point for each donor/condition was calculated as an average of the photons within a fixed region of interest (ROI) of the dorsal and ventral side of each mouse, for each mouse used (i.e., data point for 1 donor and 1 condition was an average of the measurements from 2 separate mice, for a total of 5 unique donors and 8–10 mice total per condition). Experiments were performed on 8- to 10-week-old mice that were age and sex matched within the experiments. BLI imaging was performed on an IVIS 50 (10- to 90-second exposure, bin8, field of view [FOV] 12 cm, open filter) (Xenogen). Mice were injected i.p. with d-luciferin (150 mg/kg in PBS, Gold Biotechnology) and imaged under anesthesia with isoflurane (2% vaporized in O_2_). The total photon flux (photons/second) was measured from fixed regions of interest over the entire mouse (average of dorsal and ventral images) using the Living Image 2.6 software program.

### Confocal immunofluorescence microscopy and image analysis.

For fixed cell confocal imaging, freshly isolated primary human NK cells were cocultured with K562 or HL60 target cells at a 2:1 effector/target ratio in HAB10 media with 1 ng/mL IL-15 for 30 minutes at 37°C in 5% CO_2_. Cells were gently pipetted to remove clumps and then transferred to poly-l-lysine–coated (0.01%) 8-well chambers (no. 1.5, Cellvis) for an additional 30 minutes at 37°C. After incubation, cells were fixed and permeabilized with CytoFix/CytoPerm (BD Biosciences) at 4°C for 20 minutes and then washed with PBS and permeabilized with PBSS (1× PBS, 1% BSA, 0.1% saponin). Conjugate staining was performed at 4°C overnight in PBSS using phalloidin AF555 (Life Technologies, Thermo Fisher Scientific), CD8α FITC (clone HIT8A), and perforin bv421 (clone dG9) (BioLegend). After staining, cells were washed and covered with Vectashield mounting medium (Vector Laboratories). Images were acquired using a Nikon AXR Confocal Microscope with a 60× oil immersion objective (Washington University Center for Cellular Imaging, WUCCI) on a Ti2 microscope stand, using an 8k galvo scanner. Data were exported as ND2 files for further analysis. Fiji (version 1.54) was used to process and analyze confocal images (80). After identification of NK:tumor conjugates, the *z*-slice with optimal perforin polarization and actin accumulation at the synapse was used for quantification of the fluorescence intensity of actin and CD8α. ROIs were drawn within synaptical and nonsynaptical (distal end) membrane regions of the NK cell, and the fluorescence intensity of actin or CD8α was calculated as the ROI area (μm^2^) × the MFI for each individual cell.

### Statistics.

Statistical comparisons were performed as indicated in each figure using GraphPad Prism, version 10 (GraphPad Software). Data are represented as the mean ± SEM, and all significance testing comparisons were 2 sided. Statistical tests used included 2-way ANOVA, repeated-measures 1-way ANOVA, a mixed-effects model, and a paired, 2-tailed Student’s *t* test. A *P* value of less than 0.05 was considered to be statistically significant.

### Study approval.

All animal studies were approved by the Washington University IACUC (St. Louis, Missouri, USA), and experiments were conducted with the approval of and in accordance with the guidelines of the Washington University Animal Studies Committee.

### Data and code availability.

Bulk RNA-Seq and CUT&TAG data are available in the GEO database under accession numbers GSE236394 and GSE263686, respectively. For scRNA-Seq data, healthy donor purified NK cells were used (dbGaP study accession: phs002681) (81). Values for all data points in graphs are reported in the Supporting Data Values file.

## Author contributions

CCC and TAF conceptualized the study. CCC, MMBE, TAF, and EMM designed the methodology. CCC, PW, HKD, JAF, JT, LM, MF, KH, NDM, TS, HF, MBH, AYZ, MTJ, DARG, EMM, MMBE, and JEP conducted studies and formal analysis. CCC and TAF wrote the original draft of the manuscript. All authors reviewed and edited the manuscript.

## Supplementary Material

Supplemental data

Unedited blot and gel images

Supporting data values

## Version 1 05/28/2024

In-Press Preview

## Version 2 08/01/2024

Electronic publication
