# Supplementary material for: Induced CD8α identifies human NK cells with enhanced proliferative fitness and modulates NK cell activation
Source: J Clin Invest. 2024 May 28;134(15):e173602. doi: 10.1172/JCI173602 (PMC11291271; doi:10.1172/JCI173602)
Supplement: Supplemental data [file jci-134-173602-s160.pdf]

Supplemental Methods:

Flow Cytometry Antibodies

For flow cytometry, the following Beckman Coulter antibodies were used: CD3 (clone UCHT1, CD45 (clone A96416), CD56 (clone N901), NKG2A (clone Z199.1), NKp46 (clone BAB281). The following BD antibodies were used: CD8A (clone SK1), CD16 (clone 3G8), IFN-γ (clone B27), CD107a (clone H4A3), CD57 (NK-1) CD69 (FN50), CD137 (clone 4-1BB), Ki67 (clone B56), RUNX3 (R3-5G4), ERK1/2 (pT202, pY204), AKT (pS473), STAT5 (47/Stat5, pY694), PLCγ2 (pY759), S6 (pS235/pS236). The following Biolegend antibodies were used: NKG2D (clone 1D11), NKp30 (clone P30-15). NKp44 (clone P44-8), IgG1 control (clone MG1-45). The following eBioscience antibodies were used: TNF (clone Mab11). For antibody cross-linking, the following Biolegend (LEAF™ or Ultra-LEAF™ purified) mouse IgG1 monoclonal antibodies were used: purified goat anti-mouse IgG1 (clone Poly4054), mIgG1 isotype control (clone MG1-45), CD2 (clone RPA-T2.10), CD8A (clone RPA-T8 or SK1), CD16 (clone 3G8), NKG2D (clone 1d11), CD337 / NKp30 (clone P30-15), CD335 / NKp46 (clone 9E2). The following purified mIgG1 eBioscience antibodies were used: CD224 /2B4 (clone eBioC1.7). The following purified mIgG1 NA/LE BD antibodies were used: CD226 / DNAM-1 (clone DX11).

Flow Cytometric Analysis and Sorting

Cell staining was performed by incubating cells with a master mix containing staining antibodies (and goat serum to prevent non-specific binding) for 15 minutes at 4°C in the dark, then washed twice with FACS buffer (PBS with 2% FBS, 2mM EDTA). Data were acquired on a Gallios flow cytometer (Beckman Coulter) and analyzed using FlowJo (Tree Star, version 10.8) software. Dead cells were stained using the LIVE/DEAD Fixable Near-IR Dead Cell Stain Kit (Thermofisher Scientific) according to the manufacturer's instructions, except for phospho-flow. eBio Fix/Perm was used for all intracellular staining except phospho-flow. CD8α+ and CD8α− CD56^{bright} and

CD56^{dim} NK cells were sorted to >99% purity using FACSAria II Cell Sorter (BD Biosciences). All MFI values shown are calculated using the median fluorescence intensity.

Cell lines

K562 cells (ATCC, CCL-243) were obtained from ATCC in 2008, viably cryopreserved, thawed for use in these studies, and maintained for no more than 2 months at a time in continuous culture as described (1). Prior to our studies, the K562 cells were authenticated by confirming cell growth morphology (lymphoblast), growth characteristics, and functionally as NK-cell–sensitive targets in 2014 and 2015. Cells were cultured in 'HAB10 media" - RPMI1640 supplemented with L-glutamine, HEPES, NEAA, sodium pyruvate, and Pen/Strep/Glutamine containing 10% FBS (Hyclone/GE Healthcare, Logan, UT).

NK cell purification and cell culture

Human platelet apheresis donor PBMCs were obtained by Ficoll-Paque (Cytiva Life Sciences) density gradient centrifugation. NK cells were purified using RosetteSep (StemCell Technologies, ≥ 95% CD56^{+}CD3^{-}) and used for selected experiments. NK cells were cultured in HAB10 supplemented with rhIL-15 (1 ng/mL) to support survival, with 50% of the medium being replaced every 2-3 days.

RNA Sequencing

CD8α+ and CD8α- CD56^{bright} and CD56^{dim} NK cells (CD3-, CD19-, CD14-) were sorted and stored in TRIzol at –80°C until RNA isolation using the Direct-zol RNA MicroPrep Kit (Zymo Research). NextGen RNA sequencing was performed using an Illumina HiSeq 2500 sequencer. RNA-sequencing reads were then aligned to the Ensembl release 76 primary assembly with STAR version 2.5.1a. Gene counts were derived from the number of uniquely aligned unambiguous reads by Subread:featureCount version 1.4.6-p5 and imported into the R/Bioconductor package

EdgeR5 and TMM normalization size factors were calculated to adjust for samples for differences in library size. The TMM size factors and the matrix of counts were then imported into the R/Bioconductor package Limma. Weighted likelihoods based on the observed mean-variance relationship of every gene and sample were then calculated for all samples and the count matrix was transformed to moderated log 2 counts-per-million. Differential expression analysis was then performed to analyze for differences between conditions and the results were filtered for only those genes with Benjamini-Hochberg false-discovery rate adjusted p-values less than or equal to 0.05. Single-cell sequencing and analysis was performed as described previously on publicly available datasets (2).

Functional assays to assess cytokine production

Six days following electroporation and culture in HAB10 + 1 ng/mL IL-15, control or CD8A KO NK cells were plated at approximately 2-2.5x10^5 cells/well of a round bottom 96-well plate and stimulated in a standard NK cell functional assay. Briefly, cells were incubated for 6 hours with either cytokines rh-(IL-12 [10 ng/mL], IL-15 [50 ng/mL], and IL-18 [50 ng/mL]) or K562 or HL60 leukemia targets at an effector to target (E:T) ratio of 5:1 in presence of an anti-CD107a PerCpCy5.5 antibody. For plate-bound assays, a 96-well flat bottom plate was coated with antibodies directed against NK activating receptors at 10 μg/mL in PBS overnight at 4’C. Wells were washed 2x with PBS, prior to addition of control or CD8 KO NK cells in the presence of anti-CD107a PerCpCy5.5. After 1 hour of stimulation, Brefeldin A and Monensin (GolgiStop/GolgiPlug, BD) were added, and 5 hours later the cells were stained with surface marker antibodies. Cells were fixed and permeablized (Cytofix/Cytoperm, BD) before the staining of intracellular IFN-γ and TNF. Cells were acquired on a Gallios flow cytometer and analyzed using FlowJo Version 10.8.1 (TreeStar) software.

Cytotoxicity Assays

Flow cytometry based killing assays were performed by coincubating control or CD8KO NK cells with carboxyfluorescein succinimidyl ester (CFSE)– labeled K562 or HL60 target cells at various effector to target ratios for 4 hours and assaying 7-aminoactinomycin D (7AAD) uptake as described previously (3).

Mass Cytometry

All mass cytometry data were collected on a CyTOF2 mass cytometer (Fluidigm) and analyzed using Cytobank. Mass cytometry data were analyzed using previously described methods, and sample staining and data collection was performed as previously described (1).

Western Blot

The following antibodies were purchased from Cell Signaling Technology: STAT4 (C46B10) Rabbit mAb, AKT (pan) (C67E7) Rabbit mAB, p44/42 MAPK (Erk1/2) (137F5) Rabbit mAb, β-Actin (8H10D10) Mouse mAb, Anti-rabbit IgG HRP-linked Antibody, Anti-mouse IgG HRP-linked Antibody. NK cells were analyzed for expression of STAT5, AKT, ERK, S6, and actin, as described previously (4) .

IL-15 Phosphorylation assays

Human NK cells were incubated in HAB10 media without cytokines at 37°C for 1 hour, at approximately 2x10^5 cells / well in a 96-well round bottom plate. IL-15 was added to wells at the indicated concentration for 45 minutes, after which CD8a-FITC mAb was added for an additional 15 minutes, to allow adequate staining of the epitope prior to methanol permeabilization. After incubation, cells were fixed with 4% paraformaldehyde (PFA) and incubated at room temperature

for 10 minutes. The cells were then pelleted and resuspended in cold 100% methanol and incubated at 4°C for 30 minutes. Cells were washed 3 times with FACS buffer. After washing, cells were suspended in surface antibody master mix as well as the appropriate phosphoflow antibodies and stained overnight at 4°C in the dark. The next morning, cells were washed twice and samples were acquired on a BeckmanCoulter Gallios flow cytometer and analyzed using FlowJo Version 10.8.1 (TreeStar) software.

References:

1. Berrien-Elliott MM, et al. Multidimensional Analyses of Donor Memory-Like NK Cells Reveal New Associations with Response after Adoptive Immunotherapy for Leukemia. *Cancer Discovery*. 2020;10(12):1854–1871.

2. Berrien-Elliott MM, et al. Hematopoietic Cell Transplantation Donor-derived Memory-Like NK Cells Functionally Persist after Transfer into Patients with Leukemia. *Science translational medicine*. 2022;14(633):eabm1375.

3. Wagner JA, et al. Stage-Specific Requirement for Eomes in Mature NK Cell Homeostasis and Cytotoxicity. *Cell Reports*. 2020;31(9):107720.

4. Wong P, et al. T-BET and EOMES sustain mature human NK cell identity and antitumor function. *Journal of Clinical Investigation*. 2023;133(13). https://doi.org/10.1172/JCI162530.

Supplemental Figure 1

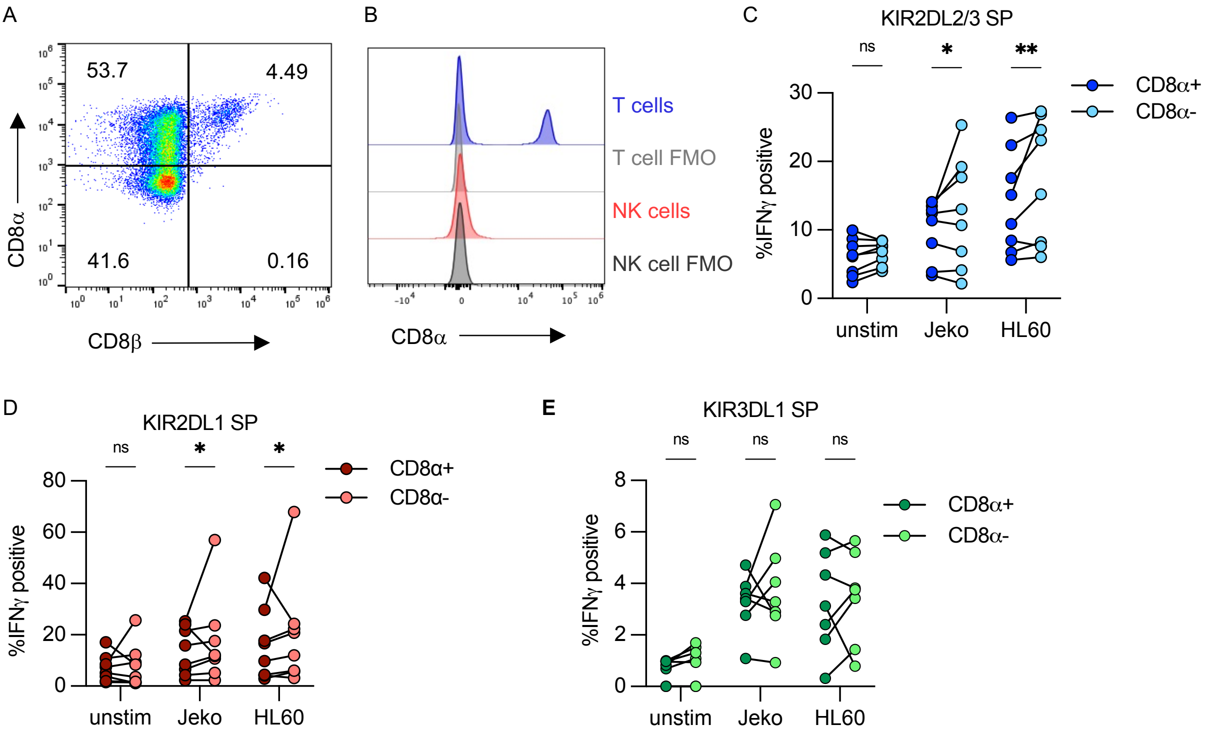

Supplemental figure 1: **CD8α is expressed on human NK cells and marks populations with distinct proliferative capacities. (A)** Representative FACS plot of human CD8α and CD8β staining on freshly isolated human peripheral blood NK cells (CD56+ CD3-). Representative staining from 1 of 4 donors. **(B)** Representative histogram of staining of mouse CD8α on T cells and NK cells from a C57/Bl6 mouse. **(C-E)** Freshly isolated NK cells were stimulated for 6 hours with Jeko-1 or HL60 cells at a 5:1 effector to target ratio, then stained for expression of IFNγ. CD8+ or CD8- NK cells were gated within CD56^{dim} NKG2A- (**C**) KIR2DL2/3 (KIR3DL1-, KIR2DL2/3+, KIR2DL1-), (**D**) KIR2DL1 (KIR3DL1-, KIR2DL2/3-, KIR2DL1+), or (**E**) KIR3DL1 (KIR3DL1+, KIR2DL2/3-, KIR2DL1-) single positive subsets. N=7-8 donors, 3 independent experiments. Statistical significance determined using two-way ANOVA with Holm-Šídák correction for multiple comparisons. *$p<0.05$, **$p<0.01$.

Supplemental Figure 2

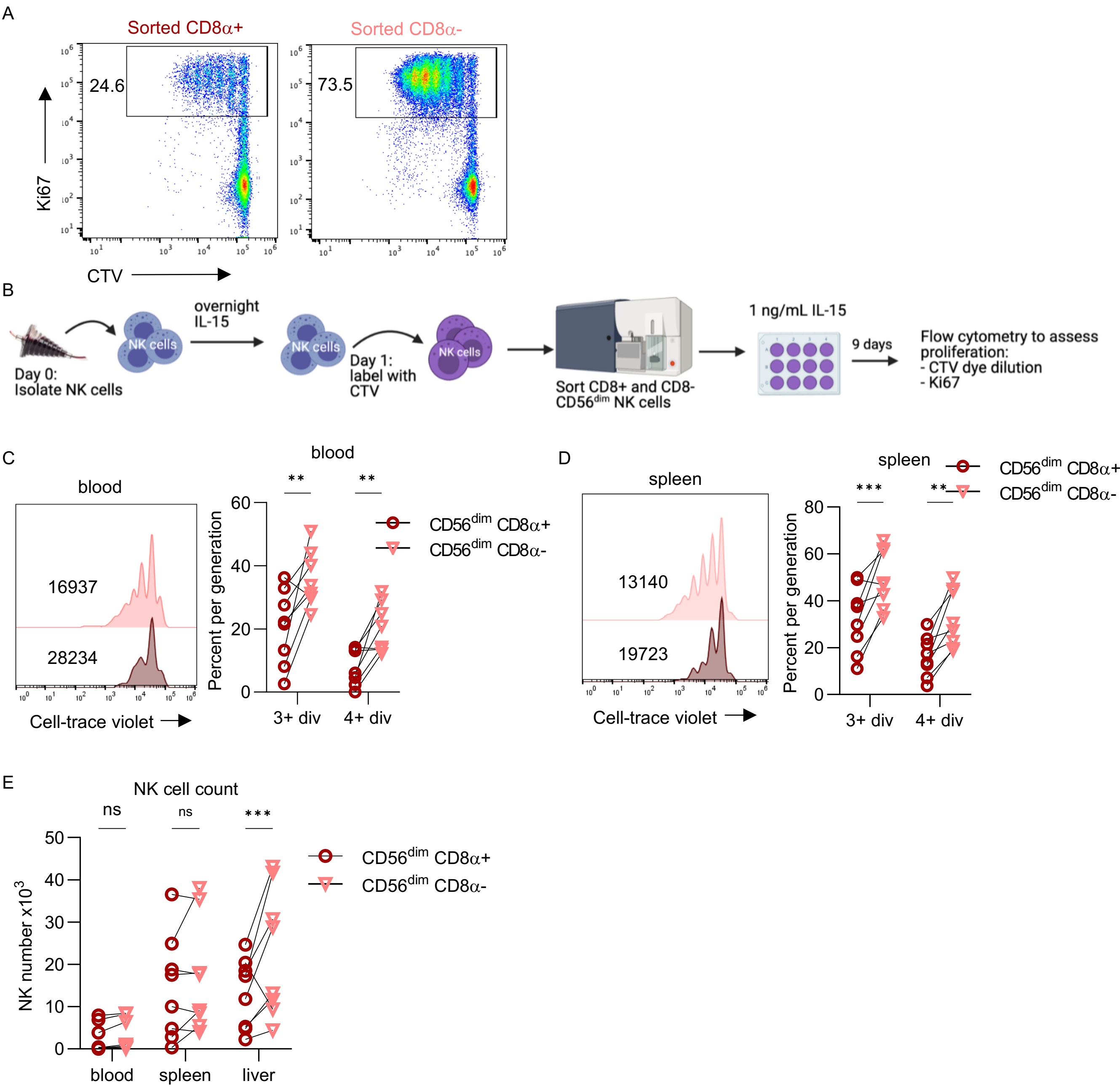

Supplemental figure 2: **CD8α marks NK cells with a distinct proliferative capacity. (A)** Representative flow cytometry plots showing gating of Ki67 on sorted CD8α+ or CD8α- NK cells, from Figure 2C. **(B)** Experimental schema for Figure 2D-E. **(C-E)** Representative histogram and summary data showing proliferation of CTV-labeled sorted CD8α+ or CD8α- CD56dim NK cells within the **(C)** blood or **(D)** spleen of NSG mice. Data shown as the percentage of NK cells that have undergone 3 or more or 4 or more divisions. **(E)** Absolute number of human CD8α+/- CD56dim NK cells recovered from the indicated organs of NSG mice at Day 9 after NK cell infusion. Number shown in blood is per mL of blood collected at time of harvest N=9 normal donors from 5 experiments. Data shown as mean +/- SEM. Statistical significance determined using two-way ANOVA with Holm-Šídák correction for multiple comparisons. *p<0.05, **p<0.01, ***p<0.001

Supplemental Figure 3

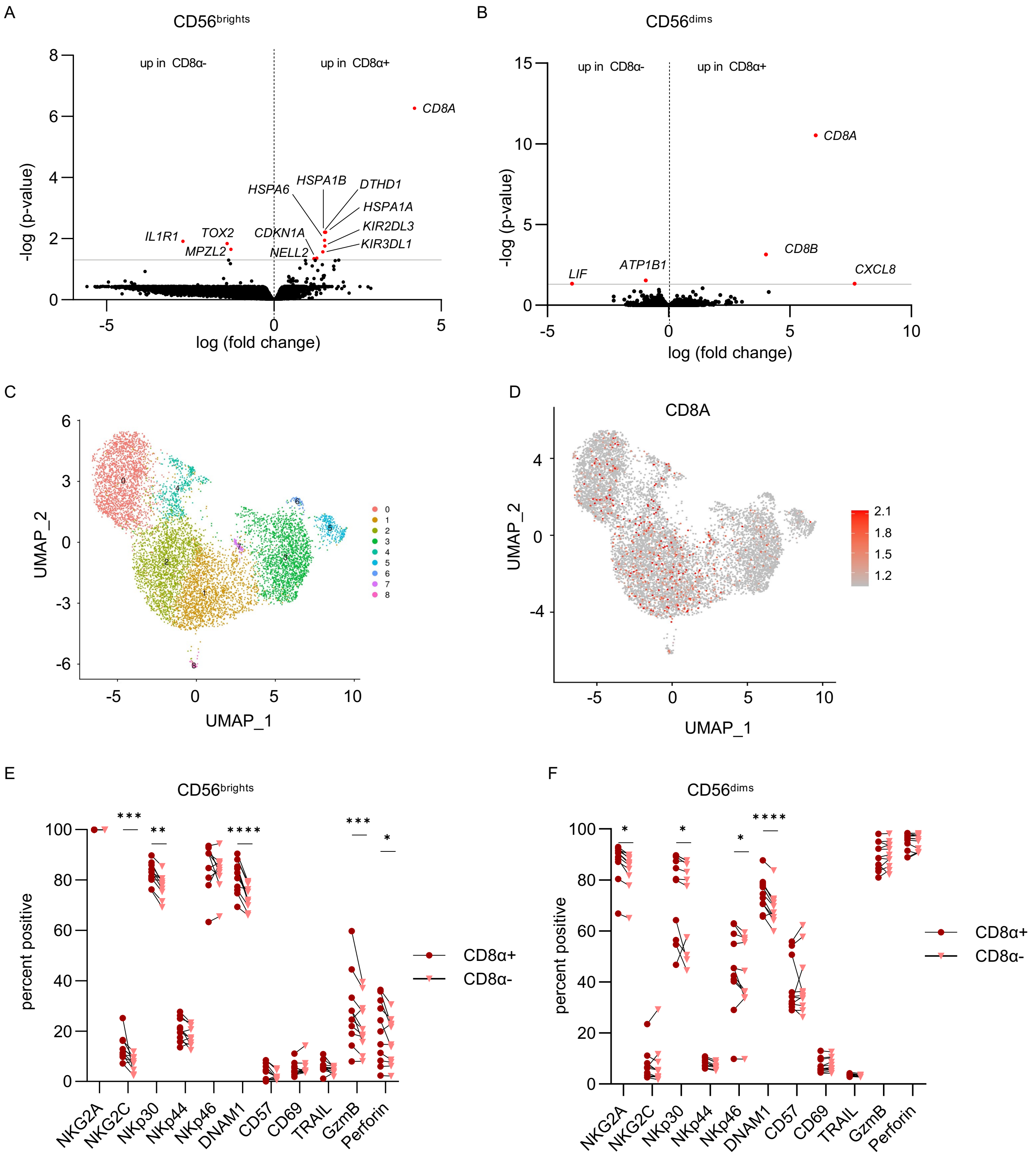

Supplemental figure 3: CD8α+ and CD8α- NK cells are transcriptionally and phenotypically similar. (A-B) Bulk RNAseq was performed on sorted CD8α+/- (A CD56[bright] or (B) CD56[dim] NK cells. Data shown as log_2(fold change) of genes expressed in CD8α- vs CD8α+. Genes that are significantly differentially expressed ($p<0.05$) are labeled in red. N=6 unique donors. **(C-D)** UMAP visualization of scRNAseq on freshly isolated healthy peripheral blood NK cells. Colors shown indicate **(C)** cluster identity, or **(D)** degree of CD8α expression across all clusters. Data shown is representative of 2 donors. **(E-F)** Mass cytometry was performed on freshly isolated NK cells from healthy donors. Data shown as the mean +/- SEM of the percentage of NK cells positive for the indicated marker, gated within CD8α+/- **(E)** CD56[bright] or **(F)** CD56[dim] NK subsets. N=11 donors. Statistical significance determined using two-way ANOVA with Holm-Šídák correction for multiple comparisons, where appropriate. *$p<0.05$, **$p<0.01$, ***$p<0.001$, ***$p<0.0001$.

Supplemental Figure 4

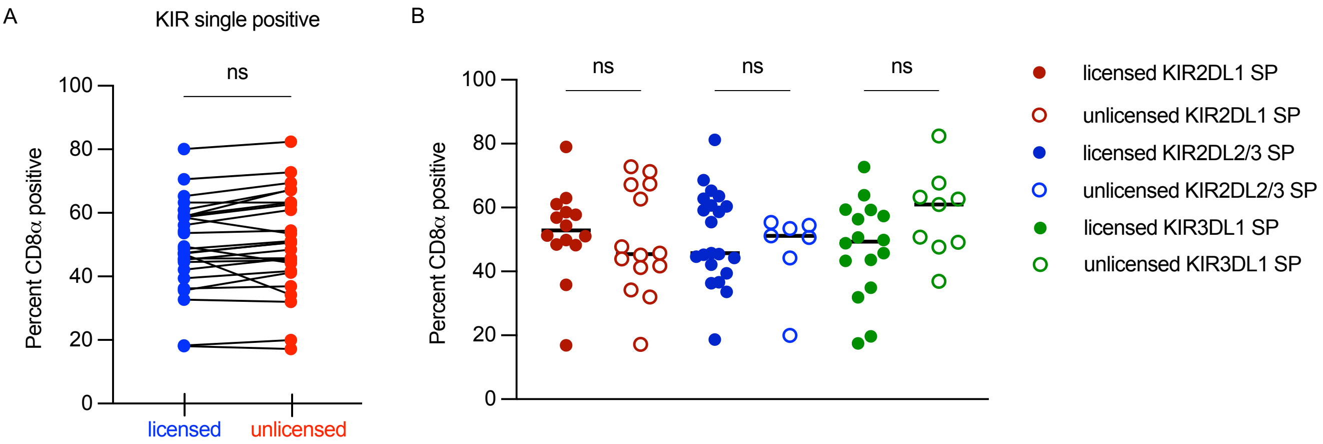

Supplemental figure 4: CD8α expression is similar on licensed and unlicensed KIR single positive NK cells. (A-B) Healthy donors were assessed for licensing status, and CD8α expression was compared within licensed (KIR2DL1+ HLA-C2+, KIR3DL1+ HLA-Bw4+, or KIR2DL2/3 HLA-C1+) or unlicensed (KIR2DL1+ HLA-C2-, KIR3DL1+ HLA-Bw4-, or KIR2DL2/3+ HLA-C1-) KIR+ CD56^{dim} NK cells. Data shown as **(A)** an aggregate within all licensed or unlicensed KIR or within **(B)** specific single positive (SP) KIR. N=28 donors. Statistical significance determined using (A) two-tailed paired t-test and (B) unpaired ordinary one-way ANOVA with Holm-Šídák correction for multiple comparisons. ns=not significant

Supplemental Figure 5

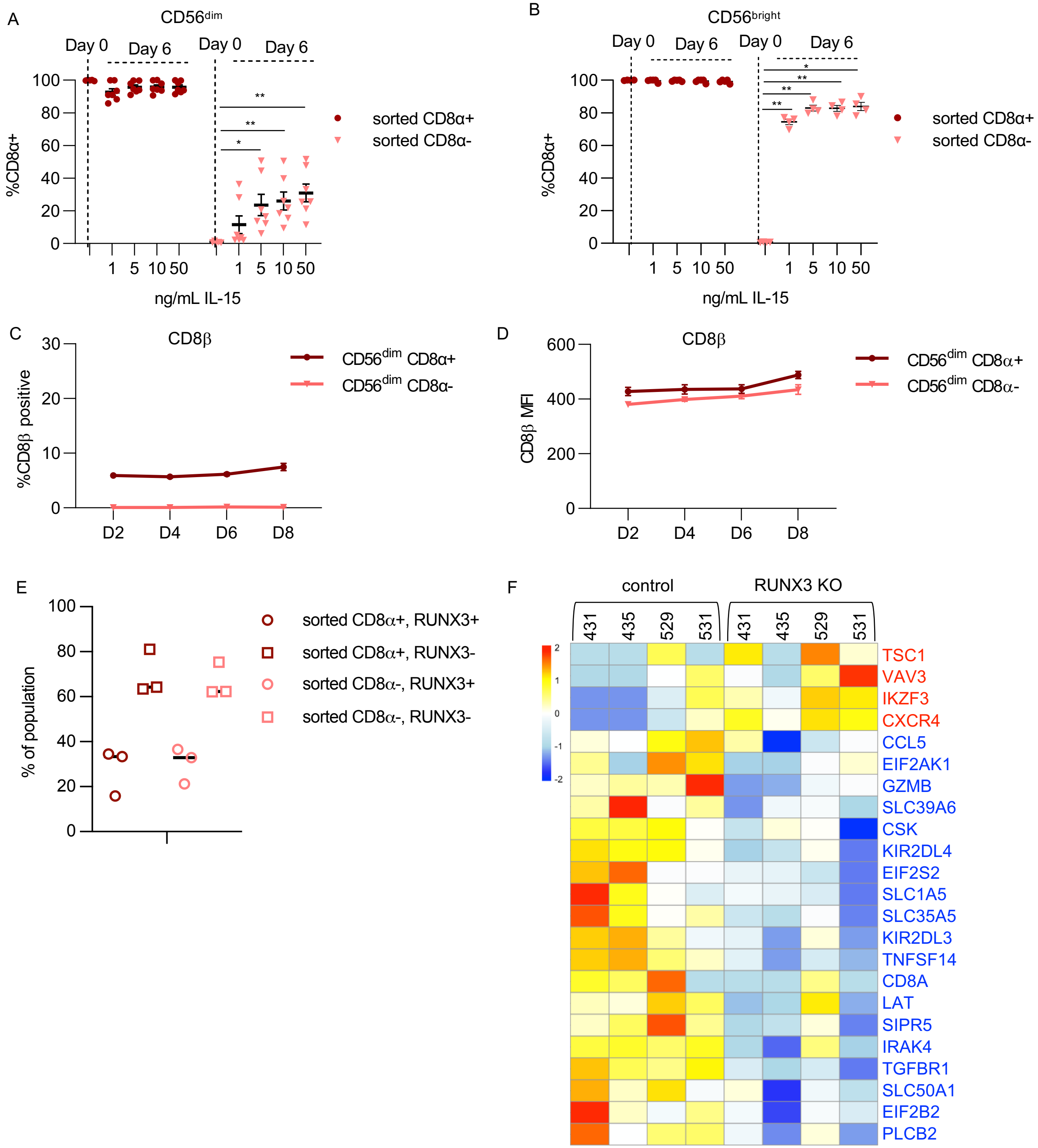

Supplemental figure 5: IL-15 induces CD8α but not CD8β expression via RUNX3 (A-B). NK cells were sorted based on CD8α expression and cultured in the indicated concentration of IL-15 for 6 days. Data shown as the percentage of NK cells positive for CD8α expression, within **(A)** CD56dim or **(B)** CD56bright NK populations. N=4-7 donors, 3 independent experiments. **(C-D)** CD56dim NK cells were sorted based on CD8α expression and cultured in 5 ng/mL IL-15 for the indicated time period (days). **(C)** Percentage of NK cells positive for CD8β expression, and **(D)** median florescence intensity of CD8β. Data shown as mean +/- SEM. N=3 donors, 2 independent experiments. **(E)** NK cells were electroporated with *RUNX3* gRNA, sorted based on CD8α expression, transferred intravenously into NSG mice, and supported with IL-15 for 9 days. Data shows the proportion of RUNX3+ or RUNX3- NK cells that were originally sorted as CD8α+ or CD8α-, within the liver of these mice, indicating a knockdown efficiency of ~60-80%. N=3 donors, 2 independent experiments. **(F)** NK cells were electroporated with *RUNX3* gRNA, cultured *in vitro* in 5 ng/mL IL-15 for 9 days, and H3K27ac was assessed via CUT&TAG. Heatmap shows the z-score normalized total H3K27ac signal for the indicated genes for each donor. See Methods for determination of statistical significance.

Supplemental Figure 6

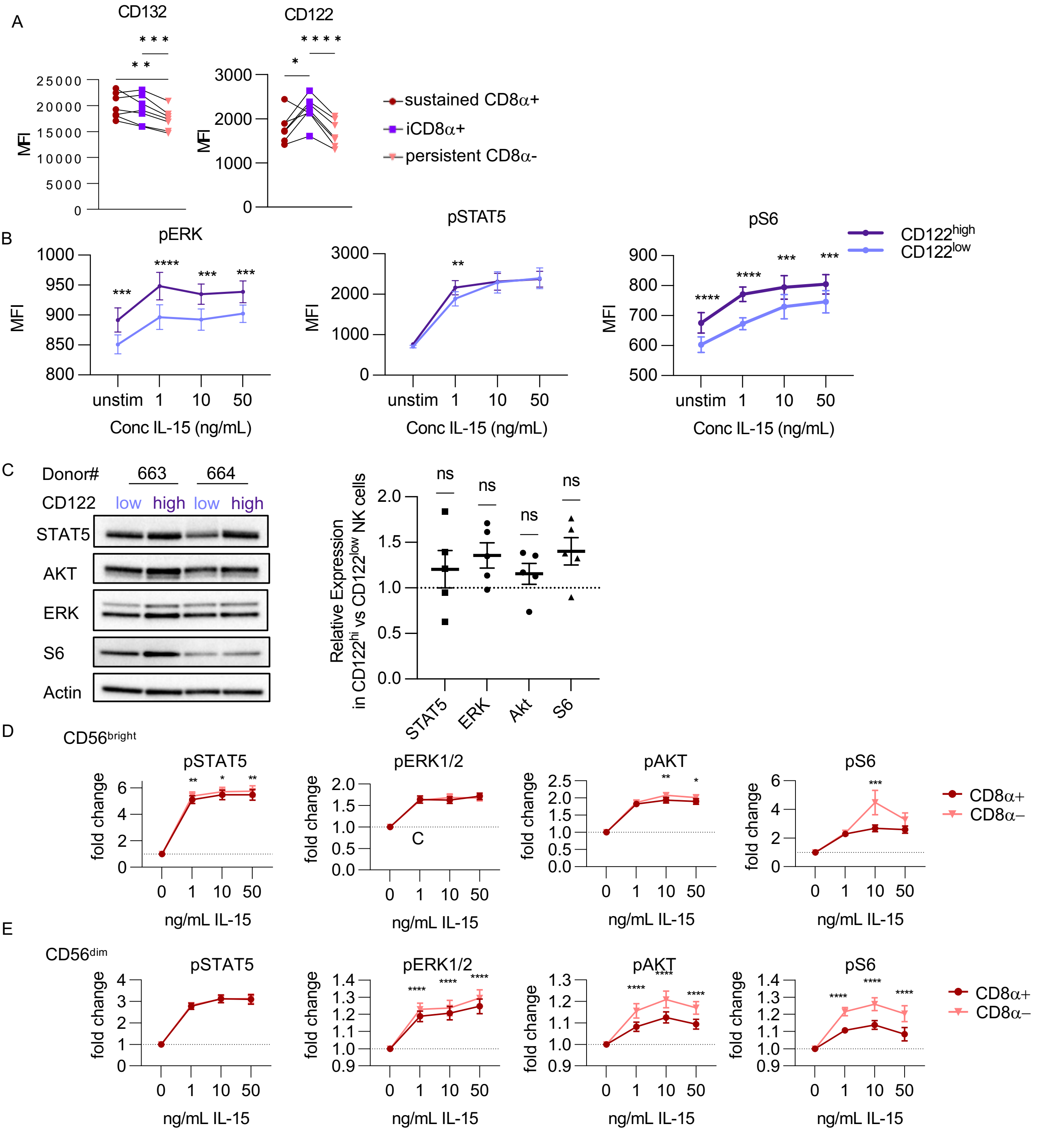

Supplemental figure 6: **Higher Il-15R expression leads to enhanced IL-15 signaling** (**A)** CD56bright NK cells were sorted based on CD8α expression, cultured in vitro in 1 ng/mL IL15 for 6 days, and stained for expression of CD132 and CD122. Data shown as the MFI within the indicated subsets. N=7 donors, 3 independent experiments. **(D-E)** Freshly isolated, unsorted **(B)** CD56bright or **(C)** CD56dim NK cells were stimulated with varying concentrations of IL-15 for 1 hour. Data shown as the fold change over the unstimulated condition within gated CD8α+/- subsets for pSTAT5, pERK1/2, pAKT, and pS6. n=13 donors, 4 independent experiments. **(B)** NK cells were rested in cytokine-free media for 1 hour, then stimulated with various concentrations of IL-15 for 1 hour. CD56dim NK cells were gated based on high or low expression of on CD122/IL-15Rβ. Data shown as the mean +/- SEM of the MFI of pERK, pSTAT5, or pS6. N=4 donors, 1 independent experiment. **(C)** NK cells were sorted based on expression of CD122/IL-15Rβ, then probed for expression of STAT5, ERK, AKT, and S6 proteins. Representative western blot and summary data showing the relative expression of the indicated marker in CD122high vs CD122low NK cells. Target protein was normalized to actin loading control of respective sample. Statistical significance determined using (A) RM one-way ANOVA, (B, D-E) two-way ANOVA (C) one sample two-tailed t-test (against hypothetical mean of 1).. ****p<0.0001, ***p<0.001, **p<0.01, *p<0.05

Supplemental Figure 7

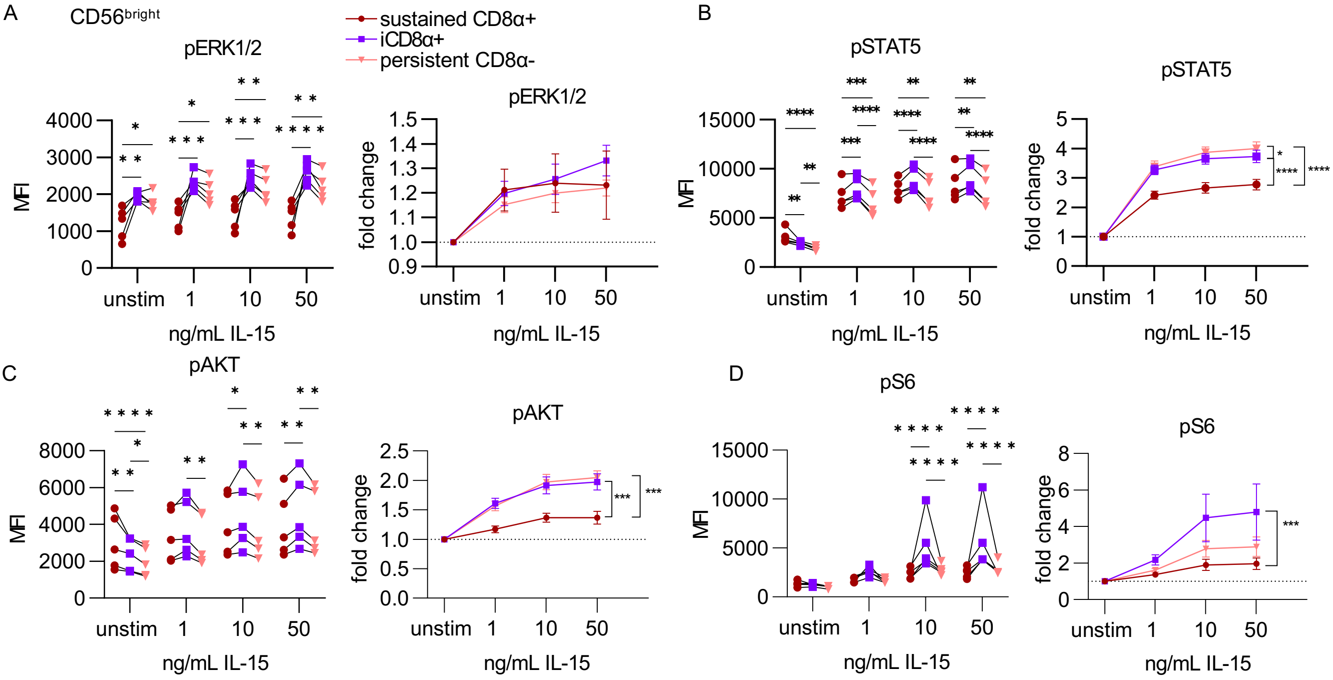

Supplemental figure 7: **Timing of CD8α acquisition corresponds to IL-15R expression and signaling** (**A-D**) CD8α+ and CD8α- CD56^{bright} NK cells were sorted and cultured for 6 days in vitro with 1 ng/mL IL-15. Cells were stimulated with the indicated concentrations of IL-15 for 1 hour. Data shown as the MFI or fold change over the unstimulated condition within the indicated subsets for **(A)** pERK1/2, **(B)**, pSTAT5, **(C)** pAKT, and **(D)** pS6. n=5 donors, 2 independent experiments. Statistical significance determined using (A) RM one-way ANOVA and (B-G) two-way ANOVA with Holm-Šídák correction for multiple comparisons. ****$p<0.0001$, ***$p<0.001$, **$p<0.01$, *$p<0.05$

Supplemental Figure 8

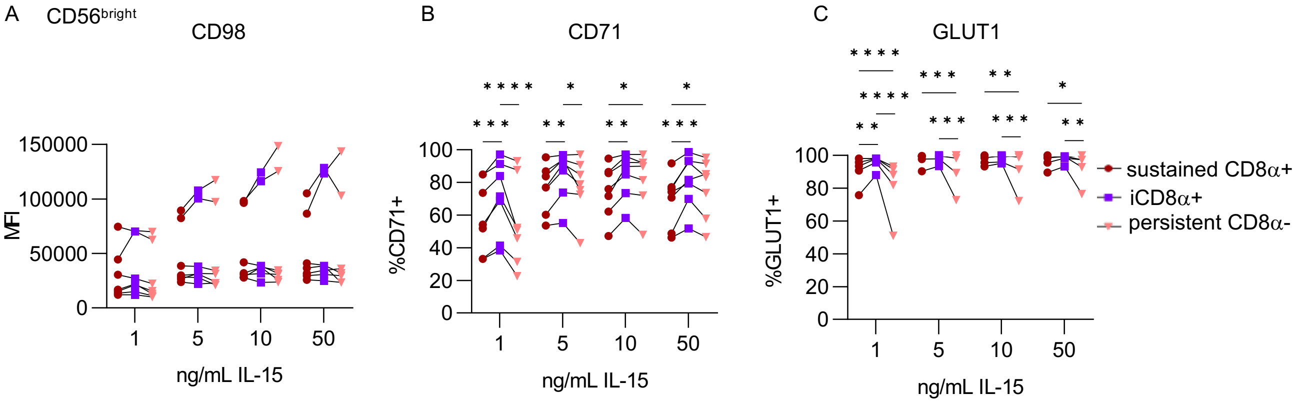

Supplemental figure 8: iCD8α+ CD56bright NK cells have modestly higher upregulation of nutrient receptors. (A-C) CD8α+ and CD8α- CD56bright NK cells were sorted and cultured for 6 days in vitro with the indicated concentrations of IL-15. Expression of nutrient receptors **(A)** CD98, **(B)** CD71, and **(C)** GLUT1 were performed via flow cytometry. N= 7 donors, 3 independent experiments. Statistical significance determined using two-way ANOVA with Holm-Šídák correction for multiple comparisons. ****p<0.0001, ***p<0.001, **p<0.01, *p<0.05

Supplemental Figure 9

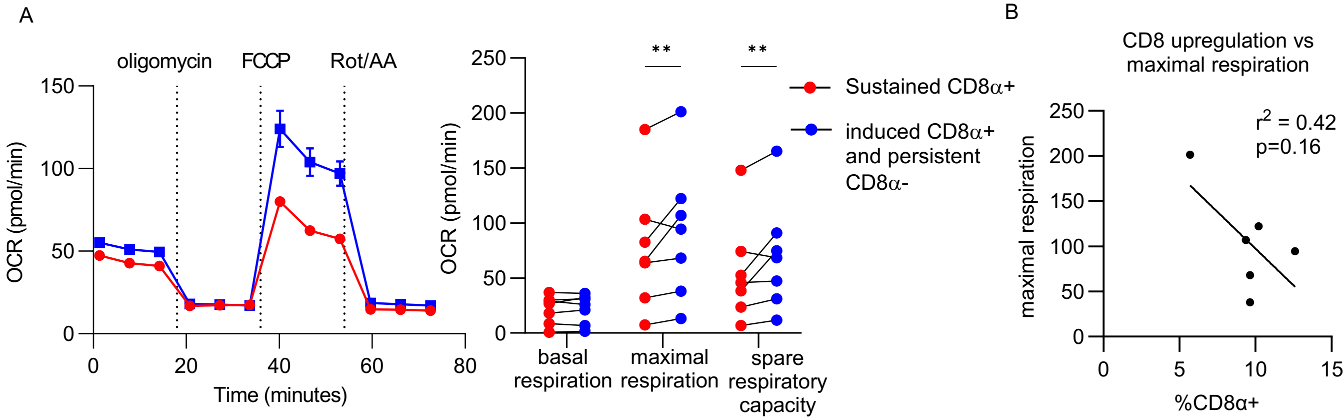

Supplemental figure 9: Sorted CD8α- NK cells have higher levels of oxidative phosphorylation. (A-B) CD8α+ and CD8α- CD56dim NK cells were sorted and cultured for 6 days in vitro with 1 ng/mL IL-15. Metabolic parameters were assessed via Seahorse using the XFe96 Extracellular Flux Analyzer. Representative donor mitochondrial stress test tracing with measurement of oxygen consumption rate (OCR) with the indicated stimulation and summary data showing basal respiration, maximal respiration, and spare respiratory capacity. N = 7 donors, 5 independent experiments. Statistical significance determined using (A) two-way ANOVA with Holm-Šídák correction for multiple comparisons and (B) Simple linear regression. *$p<0.05$, **$p<0.01$, ***$p<0.001$, ****$p<0.0001$.

Supplemental Figure 10

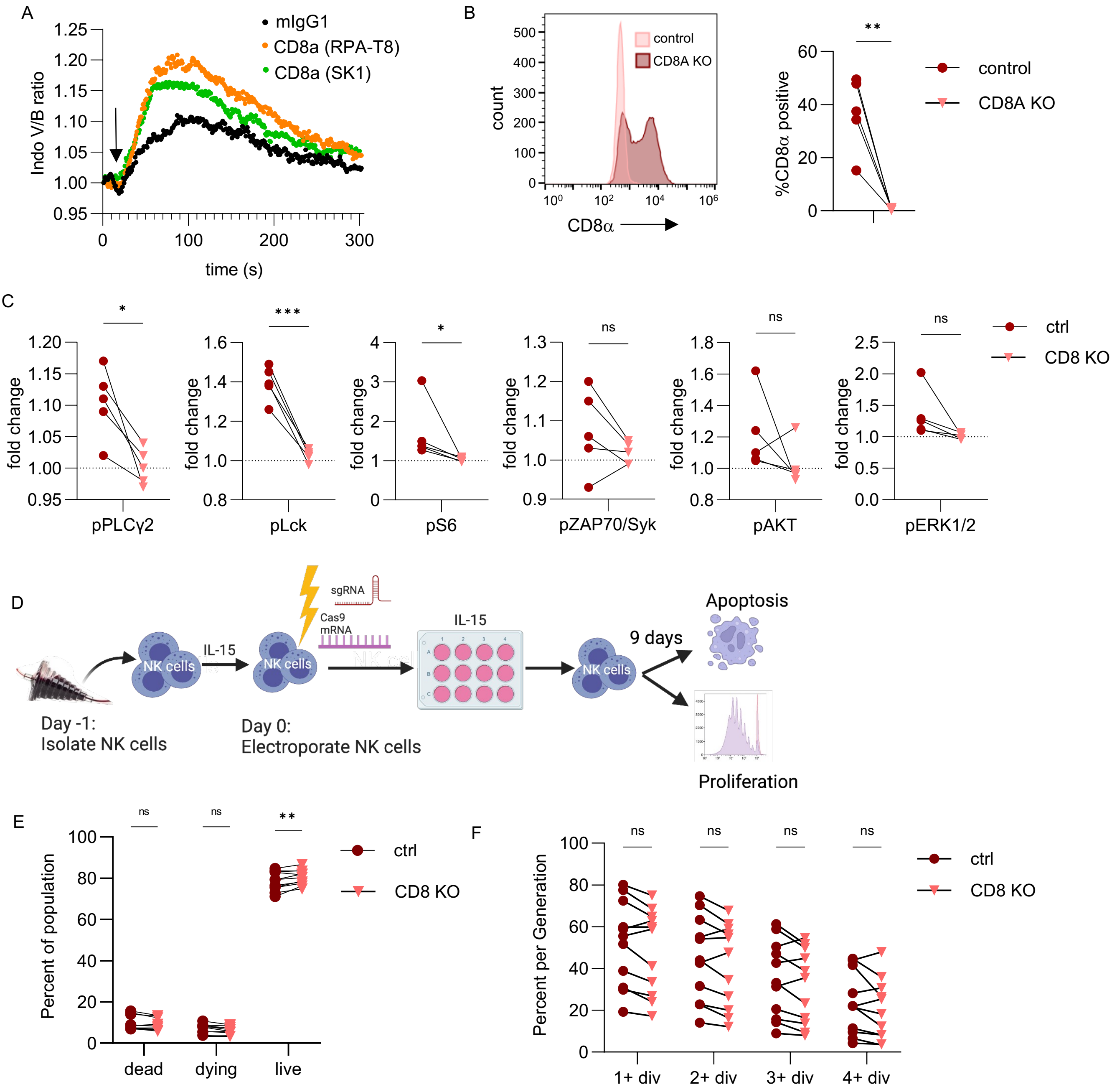

Supplemental figure 10: **CD8α ligation with antibody induces downstream signaling but does not impact NK survival or proliferation (A)** Freshly isolated NK cells were labeled with the UV-excitable calcium sensing dye Indo-1AM then incubated with the indicated monoclonal antibodies for 20 min. Ligation was induced at the indicated timepoint (arrow) with a goat anti-mouse IgG, and calcium flux was measured via flow cytometry for 5 minutes. Data shown as the normalized ratio of indo-violet (calcium-bound) over indo-blue (calcium unbound). Data shown is representative of 3 individual donors. **(B)** Representative histogram and summary data shown efficiency of CD8a knockout via crispr-cas9 electroporation. **(C)** Control or CD8A KO NK cells were ligated with anti-CD8a (clone RPA-T8) or mIgG1 isotype control for 5 minutes, and phosphorylation of the indicated signaling molecules was assessed via flow cytometry. Data shown as the fold change over mIgG1 condition, compared within control or CD8 KO cells. N=5 donors, 3 independent experiments. Statistics performed using paired two-tailed t-test. **(D-F)** Control or CD8A KO cells were labeled with cell-trace violet and cultured in 1 ng/mL IL-15 for 9 days. (**D**) Experimental schema. **(E-F)** Assessment of **(E)** viability (dead = AnnexinV+ 7AAD+, dying = AnnexinV+ 7AAD-, live = AnnexinV- 7AAD- and **(F)** Proliferation. Data shown as the percentage of NK cells within the indicated population. N=11 donors, 7 independent experiments. Statistical significance determined using (B-C) paired two-tailed t-test and (E-F) two-way ANOVA with Holm-Šídák correction for multiple comparisons. ns = not significant, *p<0.05, **p<0.01, ***p<0.001.

Supplemental Figure 11

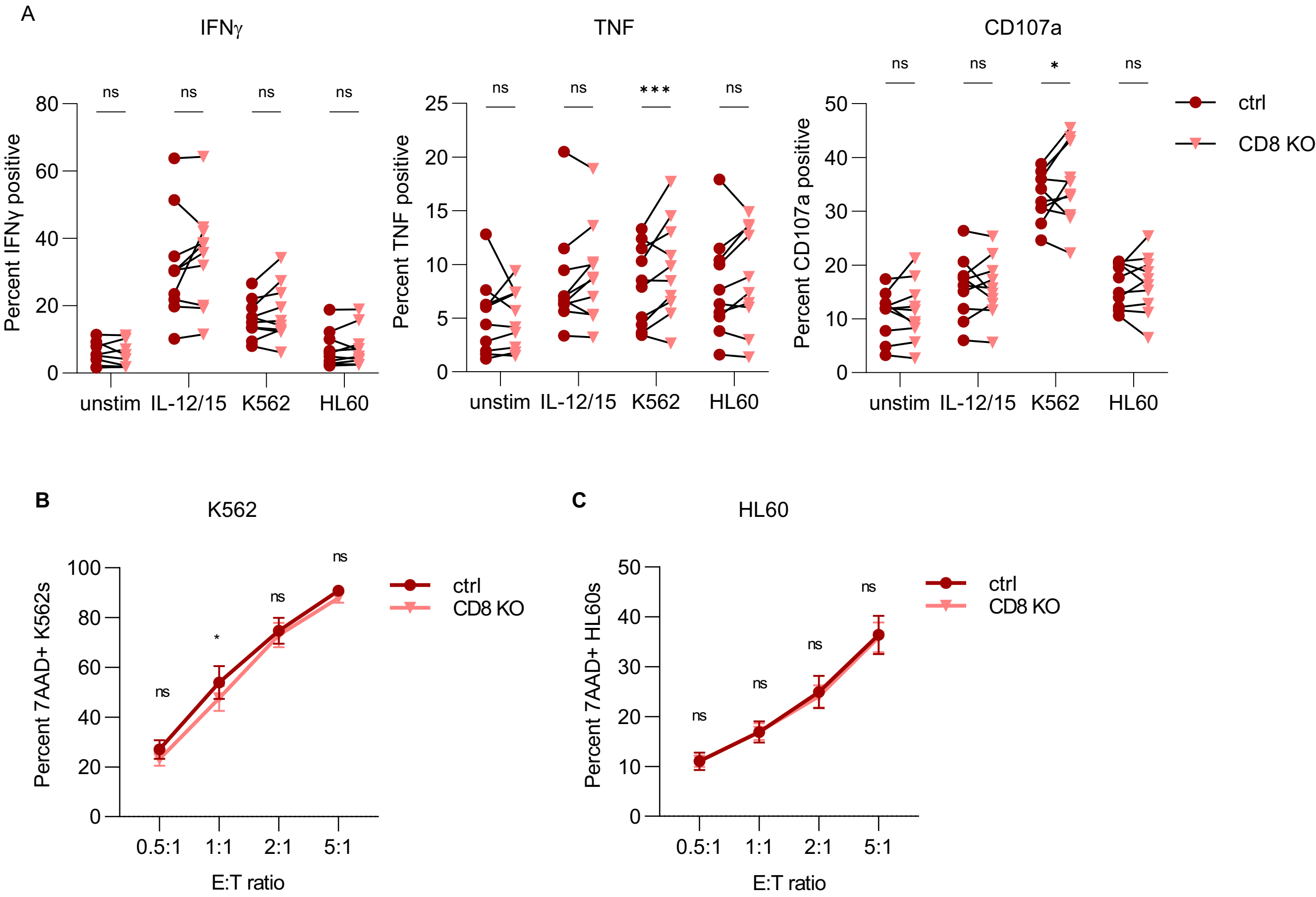

Supplemental figure 11: CD8A KO has minimal impact on cytotoxic responses to tumor or cytokine stimulation. (A-B) Freshly isolated human NK cells were electroporated with control or CD8A gRNA and cultured in vitro in 1 ng/mL IL-15 for 6 days. **(A)** control or CD8A KO cells were stimulated with IL-12 (10 ng/mL) and IL-15 (50 ng/mL), K562s (5:1 E:T), or HL60s (5:1 E:T) for 6 hours. Data shown as percentage of NK cells positive for expression of IFNγ, TNF, or CD107a. N=11 donors, 5 independent experiments. **(B)** Control or CD8A KO cells were stimulated with **(B)** K562s or **(C)** HL60s at the indicated E:T ratios. Data shown as percentage of dead (7AAD+) target cells after 6 hours. N=11 donors, 7 independent experiments. Statistical significance determined using two-way ANOVA with Holm-Šídák correction for multiple comparisons. ns = not significant, *p<0.05, **p<0.01, ***p<0.001, ****p<0.0001

Supplemental Figure 12

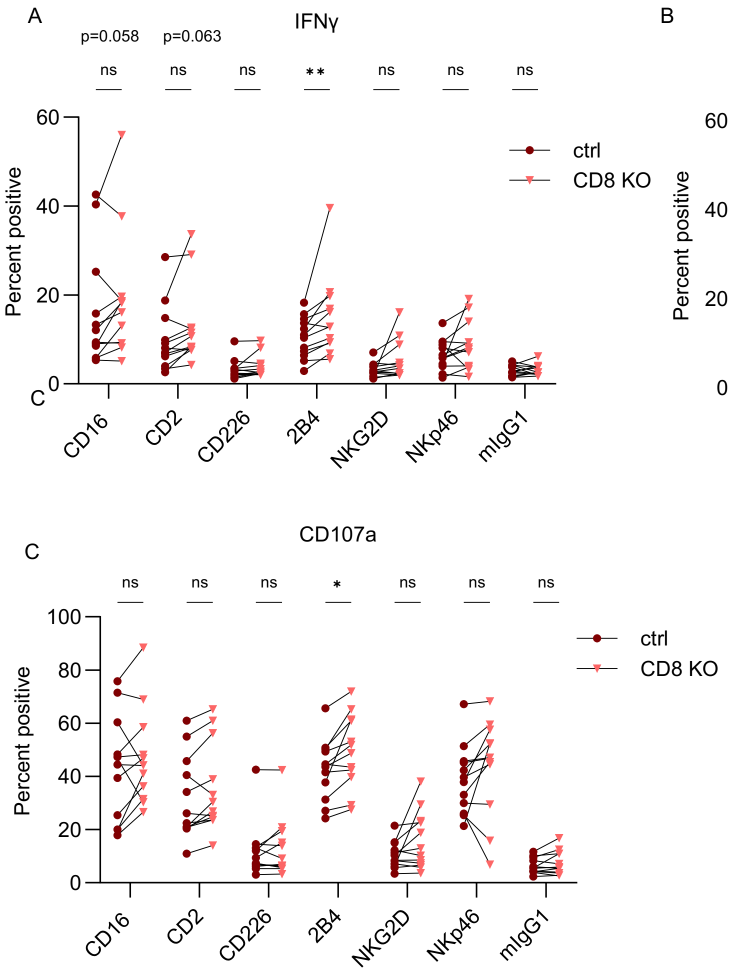

Supplemental figure 12:CD8 KO enhances cytokine secretion and degranulation following stimulation with certain activating receptors (A-C) Freshly isolated human NK cells were electroporated with control or CD8A gRNA and cultured in vitro in 1 ng/mL IL-15 for 6 days. NK cells were stimulated with plate-bound antibody (10 ug/mL) targeting the indicated activating receptors for 6 hours. Data shows the percentage of NK cells within the indicated subset positive for expression of **(A)** IFNγ, **(B)** TNF, or **(C)** CD107a. N=12 donors, 7 independent experiments. Statistical significance determined using two-way ANOVA with Holm-Šídák correction for multiple comparisons. ns = not significant, *p<0.05, **p<0.01, ***p<0.001, ****p<0.0001

Supplemental Figure 13

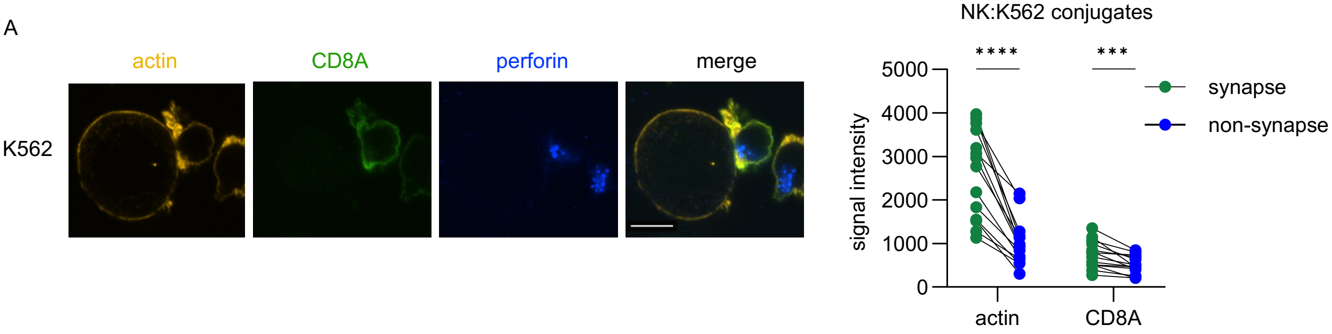

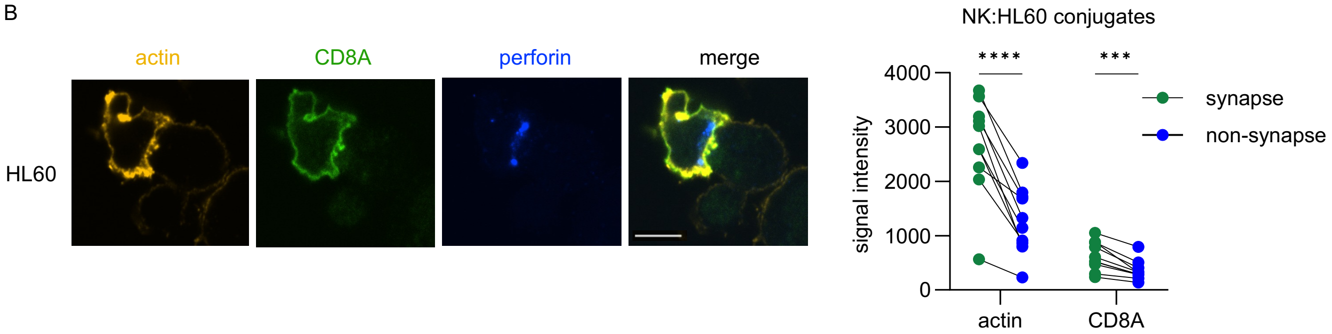

Supplemental figure 13: **CD8α is not excluded from synapses of NK:tumor conjugates (A-B)** Representative images and summary data of conjugates formed between NK cells and **(A)** K562 or **(B)** HL60 tumor cells. Data shown as the intensity of the indicated signal within synaptical or non-synaptical membrane regions. White scale bar indicates a length of 6 μm. N=5 independent experiments. Statistical significance determined using two-way ANOVA with Holm-Šídák correction for multiple comparisons. ***$p<0.001$, ****$p<0.0001$

Supplemental Figure 14

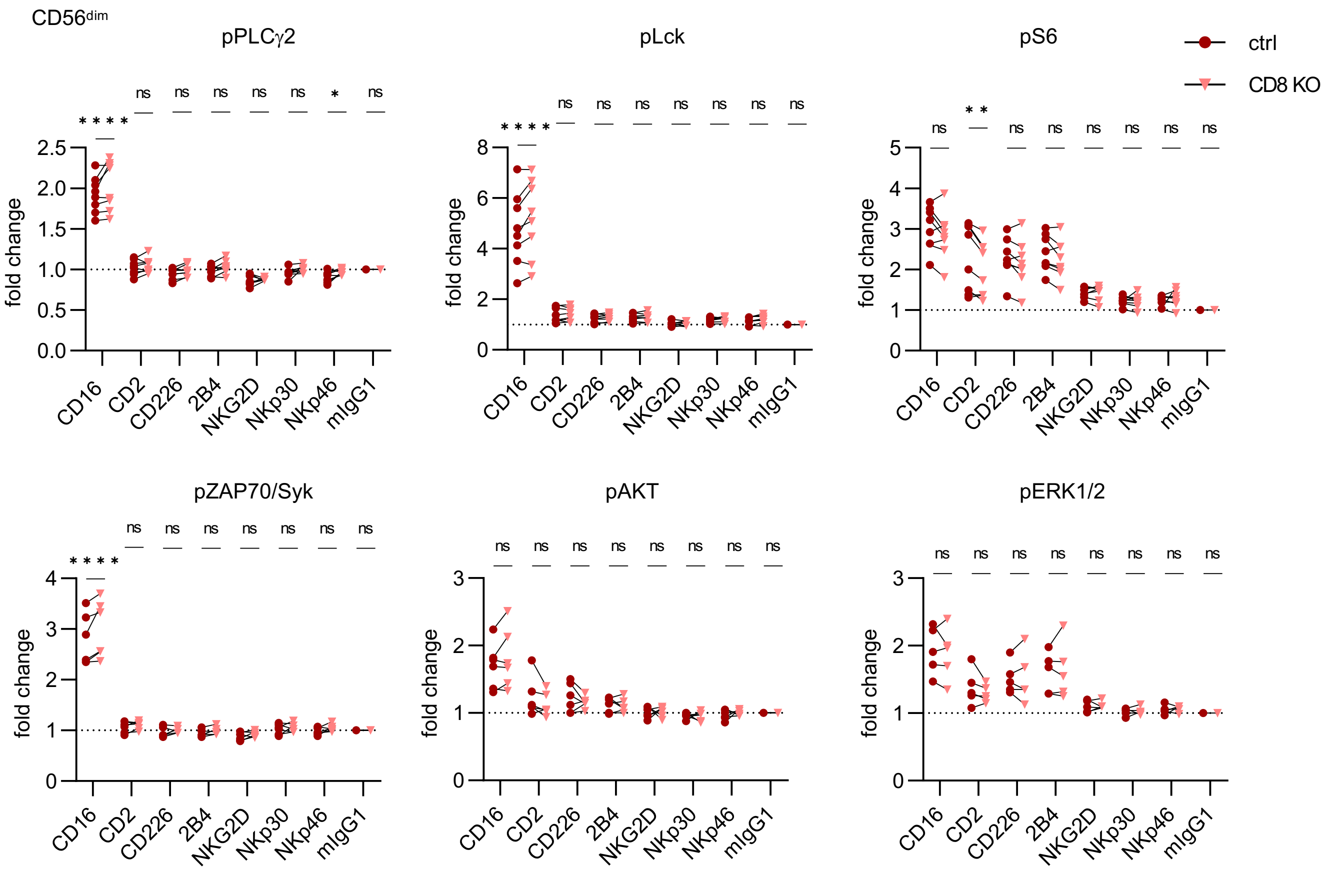

Supplemental figure 14: CD8 KO increases downstream phosphorylation following ligation of CD16 but not other activating receptors Freshly isolated human NK cells were electroporated with control or CD8A gRNA and cultured in vitro in 1 ng/mL IL-15 for 6 days. NK cells were rested in cytokine-free media for 1 hour, then incubated with 10 ug/mL of antibody against the indicated NK receptor, or mIgG1 isotype control for 20 minutes at 4ºC. Unbound antibody was washed off, and antibodies were ligated with goat anti-mouse IgG (20 ug/mL) for 5 minutes at 37ºC. Cells were fixed and permeabilized and stained for expression of pPLCγ2, pLck, pS6, pZAP70/Syk, pAKT, and pERK1/2, gated within CD56^{dim} NK cells. Data shown as the fold change over the mIgG1 treated condition. N=5-8 donors, 6 independent experiments. Statistical significance determined using two-way ANOVA with Holm-Šídák correction for multiple comparisons. ns = not significant, *p<0.05, **p<0.01, ***p<0.001, ****p<0.0001
