# Supplementary material for: Induced CD8α identifies human NK cells with enhanced proliferative fitness and modulates NK cell activation
Source: J Clin Invest. 2024 May 28;134(15):e173602. doi: 10.1172/JCI173602 (PMC11291271; doi:10.1172/JCI173602)

Full unedited gels for Supplemental Figure 6C

ERK

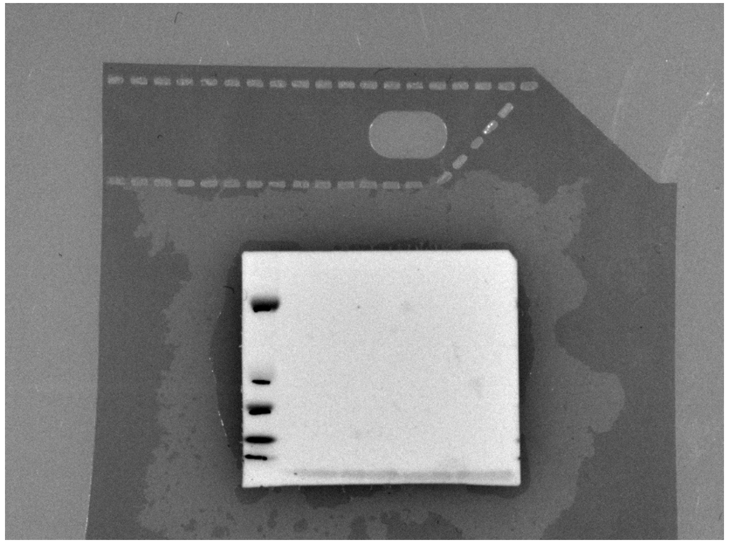

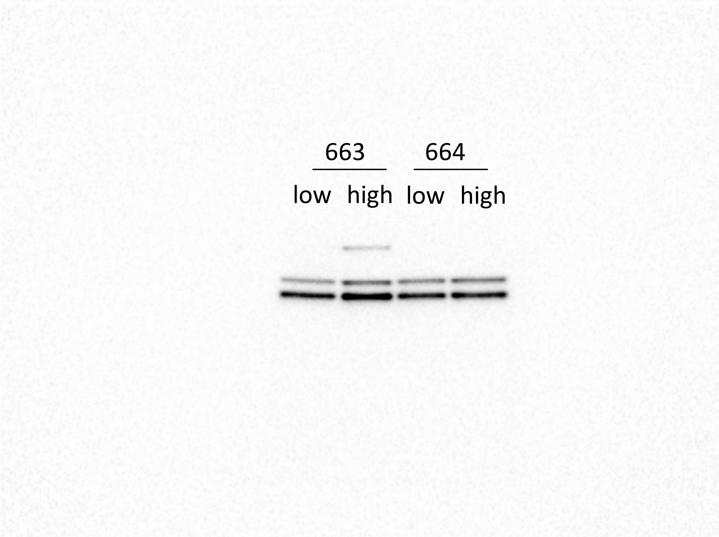

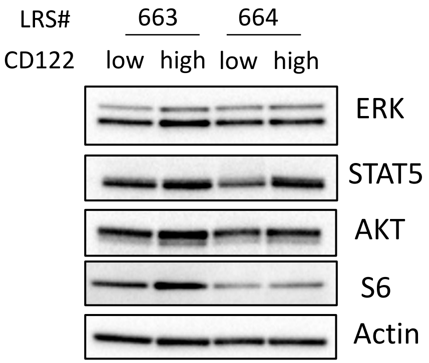

STAT5

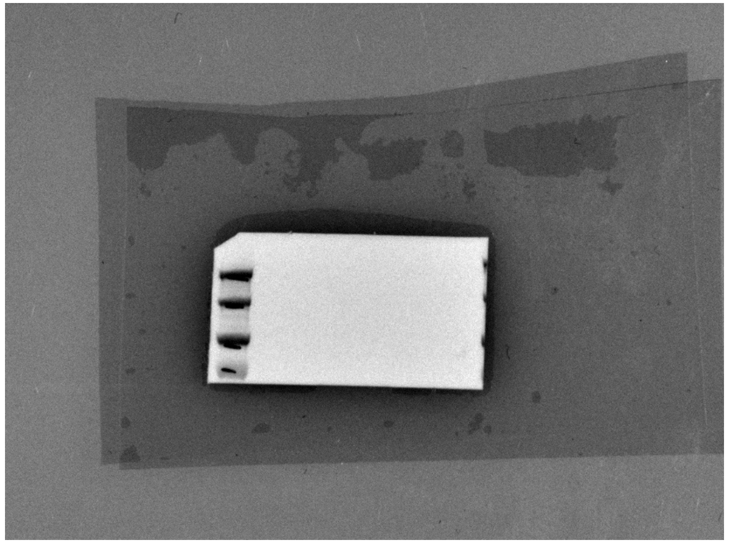

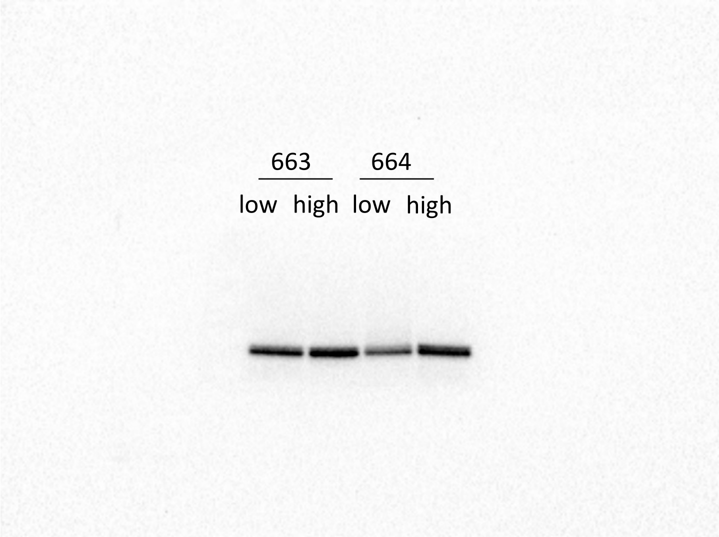

Full unedited gels for Supplemental Figure 6C

AKT

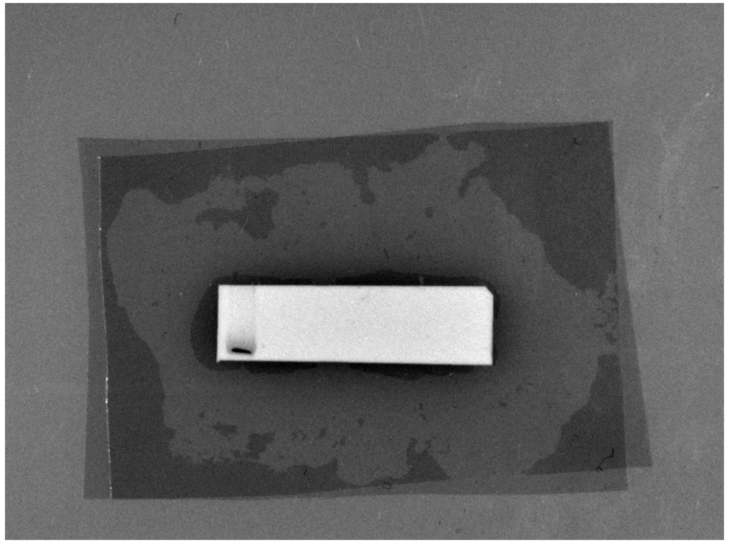

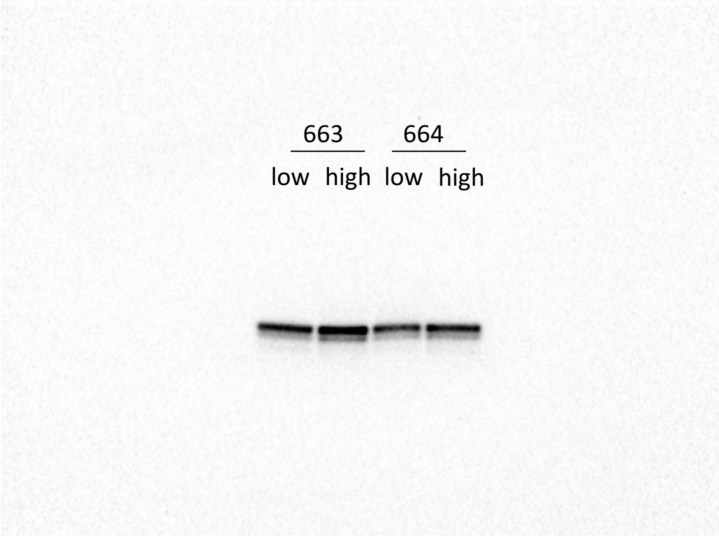

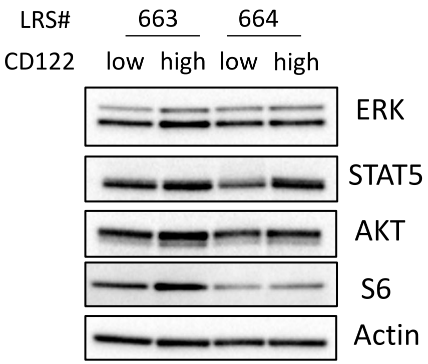

S6

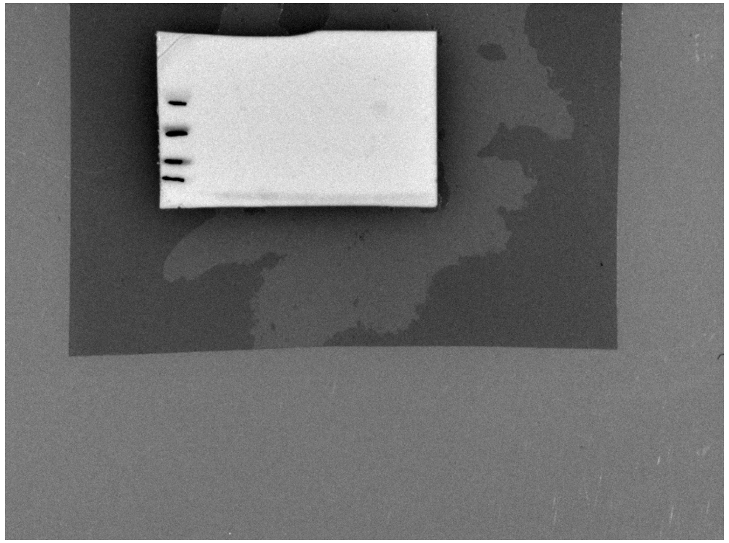

663 664

low high low high

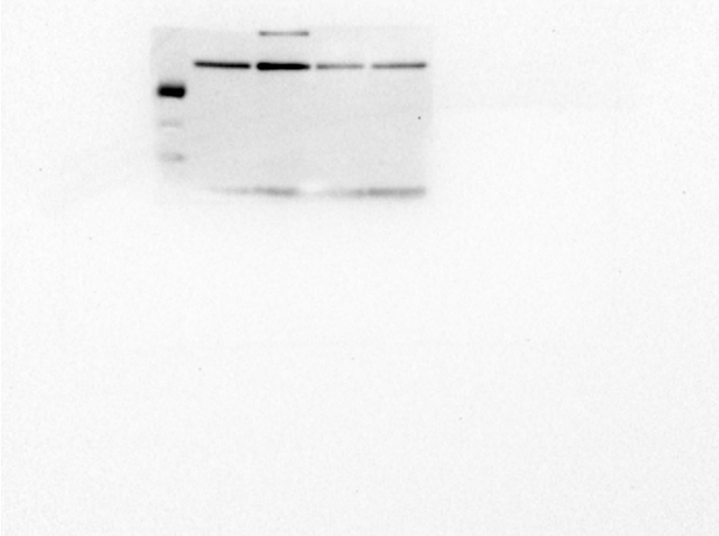

Actin

Full unedited gels for Supplemental Figure 6C

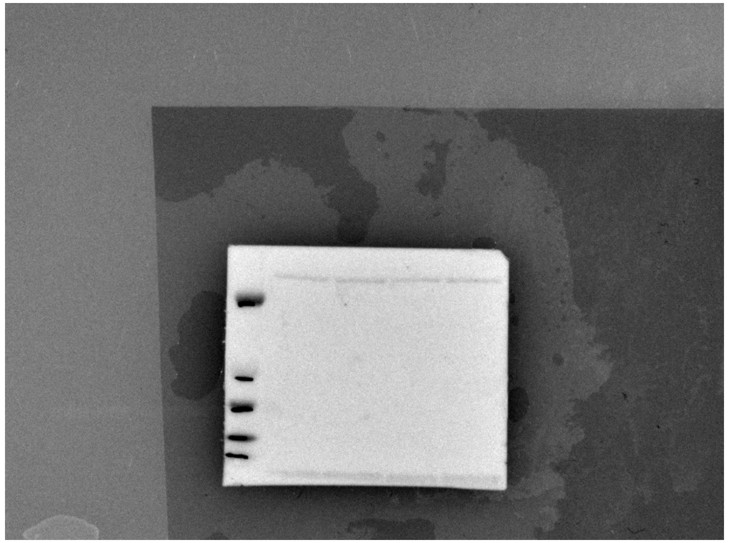

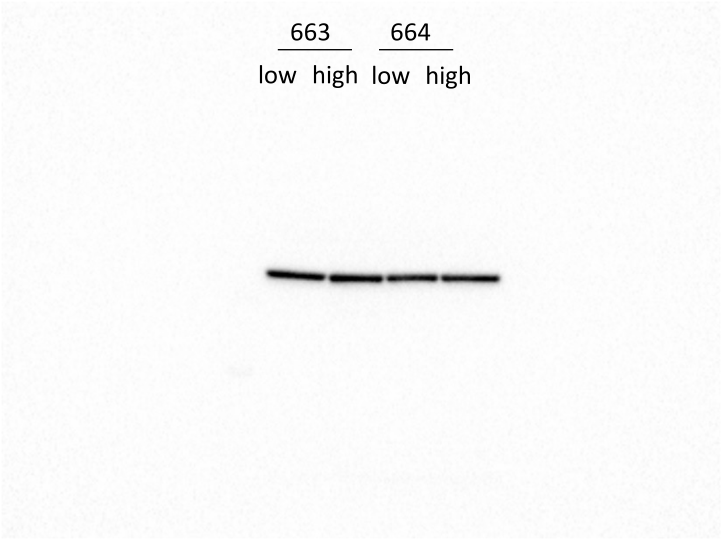

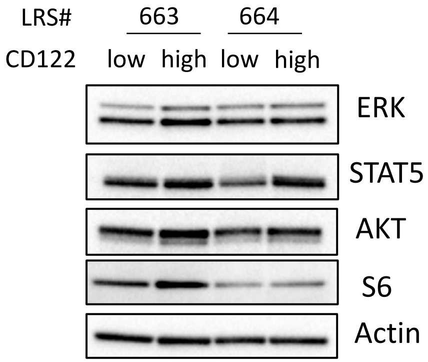

Supplement: Unedited blot and gel images [file jci-134-173602-s161.pdf]
